# Axonopathy and Reduction of Membrane Resistance: Key Features in a New Murine Model of Human G_M1_-Gangliosidosis

**DOI:** 10.3390/jcm9041004

**Published:** 2020-04-02

**Authors:** Deborah Eikelberg, Annika Lehmbecker, Graham Brogden, Witchaya Tongtako, Kerstin Hahn, Andre Habierski, Julia B. Hennermann, Hassan Y. Naim, Felix Felmy, Wolfgang Baumgärtner, Ingo Gerhauser

**Affiliations:** 1 Department of Pathology, University of Veterinary Medicine Hannover, D-30559 Hannover, Germany; Deborah.Eikelberg@tiho-hannover.de (D.E.); Annika.Lehmbecker@tiho-hannover.de (A.L.); Witchaya.Tongtako@tiho-hannover.de (W.T.); Kerstin.Hahn@tiho-hannover.de (K.H.); Andre.Habierski@tiho-hannover.de (A.H.); Ingo.Gerhauser@tiho-hannover.de (I.G.); 2 Department of Physiological Chemistry, University of Veterinary Medicine Hannover, D-30559 Hannover, Germany; Graham.Brogden@tiho-hannover.de (G.B.); Hassan.Naim@tiho-hannover.de (H.Y.N.); 3 c/o Faculty of Veterinary Science, Prince of Sonkla University, 5 Karnjanavanich Rd., Hat Yai, Songkhla 90110, Thailand; 4 Villa Metabolica, University of Mainz, Langenbeckstraße 2, D-55131 Mainz, Germany; Julia.Hennermann@unimedizin-mainz.de; 5 Department for Physiology and Cell Biology, University of Veterinary Medicine Hannover, 30559 Hannover, Germany; Felix.Felmy@tiho-hannover.de

**Keywords:** astrogliosis, axonopathy, β-galactosidase deficiency, electrophysiology, G_M1_-gangliosidosis, knockout mouse model, lipid analysis, microgliosis, neuronal vacuolation

## Abstract

G_M1_-gangliosidosis is caused by a reduced activity of β-galactosidase (*Glb1*), resulting in intralysosomal accumulations of G_M1_. The aim of this study was to reveal the pathogenic mechanisms of G_M1_-gangliosidosis in a new *Glb1* knockout mouse model. *Glb1*^−/−^ mice were analyzed clinically, histologically, immunohistochemically, electrophysiologically and biochemically. Morphological lesions in the central nervous system were already observed in two-month-old mice, whereas functional deficits, including ataxia and tremor, did not start before 3.5-months of age. This was most likely due to a reduced membrane resistance as a compensatory mechanism. Swollen neurons exhibited intralysosomal storage of lipids extending into axons and amyloid precursor protein positive spheroids. Additionally, axons showed a higher kinesin and lower dynein immunoreactivity compared to wildtype controls. *Glb1*^−/−^ mice also demonstrated loss of phosphorylated neurofilament positive axons and a mild increase in non-phosphorylated neurofilament positive axons. Moreover, marked astrogliosis and microgliosis were found, but no demyelination. In addition to the main storage material G_M1_, G_A1_, sphingomyelin, phosphatidylcholine and phosphatidylserine were elevated in the brain. In summary, the current *Glb1*^−/−^ mice exhibit a so far undescribed axonopathy and a reduced membrane resistance to compensate the functional effects of structural changes. They can be used for detailed examinations of axon–glial interactions and therapy trials of lysosomal storage diseases.

## 1. Introduction

G_M1_-gangliosidosis is a lysosomal storage disease belonging to the sphingolipidoses caused by β-galactosidase (*Glb1*) deficiency [1]. In addition to humans, it is described in cats [2,3,4,5,6,7,8], dogs [9,10,11,12], sheep [13,14,15,16], cattle [17,18], emus [19,20], American black bears [21], and different mouse models [22,23,24]. In humans, three different forms are distinguished depending on the age of onset, the clinical course, and affected organs. The infantile form (type 1) (OMIM# 230500) causes psychomotor disorders and seizures, damage to the central nervous system (CNS), hepatosplenomegaly, skeletal malformations, and early death during the first year of age. The late infantile or juvenile form (type 2) (OMIM#230600) manifests between seven months and three years of age, also with CNS and psychomotor symptoms and seizures. However, this form progresses more slowly than the infantile one. The adult form (type 3) (OMIM# 230650) emerges late, and causes CNS disturbances including speech and walking disorders, as well as skeletal malformations [25,26].

The disease develops due to lysosomal accumulation of the ganglioside G_M1_ and other lipids in the CNS and other tissues [26]. Storage is caused by a non-functional, lysosomal β-galactosidase due to numerous different mutations in humans [25,26,27,28,29] and other species like dogs [11]. Lysosomal accumulation of the ganglioside seems to lead to neuronal apoptosis and endocytoplasmic stress response [30,31], as well as axonal transport impairment in combination with loss of myelin [32]. Moreover, a neuronal and oligodendroglial miscommunication is also discussed [33]. Furthermore, a mitochondrial disorder due to autophagic processes was discussed in a *Glb1* knockout mouse model [34]. Apoptosis in a murine model of G_M1_-gangliosidosis can be caused by an accumulation of G_M1_-ganglioside in the glycosphingolipid-enriched microdomain fractions of mitochondria-associated endoplasmic reticulum (ER) membranes. This leads to an influx of Ca^2+^ originating from the ER into mitochondria, and an activation of the mitochondrial apoptotic pathway due to a Ca^2+^ overload of this organelle [35]. Neuroinflammatory changes with increased MHC class II expression, infiltration of CD4^+^ and CD8^+^ cells, elevated cytokine levels (TNFα, IL1β and TGFβ1), alterations in the blood–brain barrier permeability and apoptosis have been described in other mice affected by G_M1_-gangliosidosis [36]. Additionally, neuroinflammation results in the release of chemokines including stromal-cell-derived factor 1 (SDF-1), macrophage inflammatory protein 1-α (MIP-1α) and MIP-1β attracting peripheral immune cells [37].

Gangliosides represent sphingolipids composed of oligosaccharides connected to ceramide [38]. Their synthesis starts in the endoplasmic reticulum (ER) and continues in the Golgi apparatus. Subsequently, they are transported to the outer layer of the plasma membrane primarily via vesicle-mediated pathways [39,40]. They take part in signal transduction, cell adhesion, cell proliferation, cell differentiation, cell recognition, apoptosis, and regulation of the cytoplasmic and intranuclear calcium homeostasis [41,42]. The breakdown of G_M1_-ganglioside occurs on intra-endosomal and intra-lysosomal membranes and starts with the enzyme β-galactosidase degrading G_M1_-ganglioside to G_M2_-ganglioside. Further degradation steps produce G_M3_, lactosylceramide, glucosylceramide, ceramide, and sphingosine [43]. In mice, an increased alternative degradation pathway of G_M1_ mediated by the murine neuraminidase is described, leading to an increment of G_A1_ accumulation in *Glb1*-deficient (*Glb1*^−/−^) mice compared to humans [22]. Thus, murine neuraminidase seems to have a higher affinity towards G_M1_ compared to the human neuraminidase [22]. Different degradation pathways of gangliosides in mice are also known from Tay–Sachs mouse models [44,45].

The progressive lysosomal accumulation of periodic acid-Schiff (PAS) positive material causes cytoplasmic vacuolation, mainly of neurons. In the early onset forms of human G_M1_-gangliosidosis, storage material is also described in the liver, spleen, lung, heart valves, intestine, thymus, lymph nodes, and bone marrow [46]. Destruction of neuronal tissue in infantile and late infantile G_M1_-gangliosidosis is characterized by neuronal cell death, including axonal damage and demyelination with oligodendrocytic loss accompanied by astrogliosis and microgliosis [26,32,47]. Demyelination was also described in Alaskan Huskies [48] and cats [49,50]. As pathomechanisms for demyelination in lysosomal storage diseases, oligodendrocytic malfunction, disturbance of neuronal–oligodendroglial communication, and neuronal and axonal loss have been discussed [46].

Substrate reduction therapy, chaperone-based therapy [51,52,53,54], bone marrow transplantation [55], enzyme replacement [56], and symptomatic and supportive treatment are currently available treatment strategies for lysosomal storage diseases in humans [57]. Gene therapy certainly provides the most promising results when applied early enough but has predominantly been studied in animal models yet so far [49,50,58,59,60,61,62]. Despite characterization of several animal models and detailed studies in human patients, various aspects of the pathogenesis of murine G_M1_-gangliosidosis, particularly regarding functional aspects, axonal damage and lack of demyelination partly remain uncertain.

Different mouse models for G_M1_-gangliosidosis have been generated and investigated previously [22,23,24]. Hahn et al. (1997) created *Glb1* knockout mice by inserting a neomycin resistance gene into the middle of exon 6 [22]. Another *Glb1* knockout mouse model was created by Matsuda et al. (1997) by inserting a neomycin resistance cassette into exon 15 [23]. Przybilla et al. (2019) targeted exon 8 of the murine *Glb1* to generate a knockout mouse model [24]. All mouse models developed lesions characteristic of G_M1_-gangliosidosis [22,23,24]. Furthermore, feline models have been utilized in therapy trials, particularly gene therapy [49,50]. The mouse models were classified as [23] or compared with [22,24] the infantile or juvenile form of G_M1_-gangliosidosis despite the late onset of the disease. Moreover, myelin changes have not been described so far. The aim of the present study was to analyze the development of clinical signs, histological and immunohistochemical changes with special emphasis on axonopathy, lipid metabolism and associated electrophysiological changes in a new murine model created by an innovative gene targeting approach. Gaining a better understanding of this lysosomal storage disease will facilitate the development of innovative treatment strategies in the future.

## 2. Materials and Methods

### 2.1. Animals

Animals were housed in individually ventilated cages (Tecniplast Deutschland GmbH, Hohenpeißenberg, Germany) with 12 h light and 12 h darkness at 22–24 °C and 50%–60% humidity. Food for maintenance and breeding (ssniff Spezialdiäten GmbH, Soest, Germany) as well as water were provided ad libitum. Enrichment of the cages included mouse houses (Tecniplast Deutschland GmbH) and nesting material (ssniff Spezialdiäten GmbH).

### 2.2. Generation of Transgenic Mice

*Glb1*^−/−^ transgenic mice were generated by inserting a 636 bp fragment of a lacZ gene into the murine *Glb1* exon 15. Transcription activator-like effector nucleases (TALENs) and a knock-in vector, constructed by the company Eurofins Genomics GmbH, Ebersberg, Germany, was used to insert the fragment into the genome of murine oocytes of C57BL/6 mice in cooperation with the company Cellectis SA, Paris, France and the Laboratory of Transgenic Models of Diseases from the Institute of Molecular Genetics of the ASCR v.v.i. (Prague, the Czech Republic). The validation of the insert in exon 15 of the murine *Glb1* gene (NM_009752.2, GeneID: 12091) after creating the *Glb1*^−/−^ mice was performed using standard PCR methods with the forward primer GLB1TG_1_MI14 (5′-CAG CAC ACT CCT TAA ATC TCA GC-3′) in intron 14, the reverse primer GLB1TG_10_MI15 (5′-CTC AGT ACC GAG AAG GAA TCA G-3′) in intron 15 and Phusion^®^ HF DNA-Polymerase (New England Biolabs GmbH, Frankfurt, Germany). Gel electrophoresis revealed heterozygous animals for the *Glb1* knockout, which were further examined by cloning the transgene amplicon into a pCR^®^ 4-TOPO^®^ Vector (Invitrogen™, Life Technologies Ltd., Paisley, UK). To generate the TA-overhang necessary for the TOPO-cloning, a second PCR with 30 cycles using the Advantage^®^ HF PCR Kit (Takara Bio Europe/Clontech S.A.S., Saint-Germain-en-Laye, France) was performed using the same primers. Ligation of the segment in the pCR^®^ 4-TOPO^®^ Vector was performed by using the TOPO TA cloning kit (Invitrogen™). One clone per *Glb1*^−/−^ mouse was sequenced by using the primers M13 forward (5′-GTA AAA CGA CGG CCA G-3′) and M13 reverse (5′-CAG GAA ACA GCT ATG AC-3′). Sequences were analyzed using CLC Main Workbench 7 (CLC Bio, Qiagen GmbH, Hilden, Germany).

### 2.3. Genotyping of Mice

DNA was extracted from ear punch tissue taken from three-week-old mice. Digestion of the tissue was performed with digestion buffer 5 mM Ethylenediaminetetraacetic acid (EDTA), pH 8.0; 200 mM sodium chloride (NaCl), 100 mM 2-Amino-2-(hydroxymethyl) propane-1,3-diol (Tris), pH 8.0; 0.2% sodium dodecyl sulfate (SDS); 0.4 mg proteinase K/mL (Carl Roth GmbH & Co. KG, Karlsruhe, Germany) overnight. Standard genotyping of the mice was performed using PCR with the forward primer *Glb1* Primer 0 fwd (5′-CTG TTG GCT TGA GAC CAG TGT AGT C-3′) binding in intron 14 and the reverse primer *Glb1* Primer 0 rev (5′-GAT GCA TAC CTT GGA CCA CCC AG-3′) binding in exon 15 of the *Glb1* gene. Subsequently, gel electrophoresis was performed in a 2% ethidium bromide gel for visualization of the PCR fragments.

### 2.4. Cell Culture of Fibroblasts

Murine fibroblasts from the subcutis of the abdomen and thorax of *Glb1*^−/−^ and wildtype mice were isolated during necropsy as previously described [63,64] and used for the analysis of *Glb1* mRNA and a β-galactosidase enzyme assay [48,65]. For this purpose, explants from the subcutis were transferred to petri dishes (Nunc GmbH, Wiesbaden, Germany) and cultured in high glucose Dulbecco’s Modified Eagle Medium (DMEM, Gibco^®^, Thermo Electron LED GmbH, Langenselbold, Germany) with 30% fetal calf serum (FCS) and 1% penicillin/streptomycin at 37 °C and 5% CO_2_. At 80% confluency, cells were passaged and used for further analysis.

### 2.5. Analysis of Glb1 mRNA

*Glb1* mRNA was isolated from cultured murine *Glb1*^−/−^ fibroblasts using TRIzol™ Reagent (ThermoFisher Scientific Inc., Carlsbad, CA, USA) in accordance with the manufacturer’s protocol. Reverse transcription was performed with random primers (Promega GmbH, Mannheim, Germany) and the Omniscript^®^ Reverse Transcription Kit (Qiagen GmbH) following the manufacturer’s instructions. PCR of resulting cDNA was accomplished with the forward primer p864_*Glb1*_Exon14_Mus_S (5′-TGG TGG AGA ACA TGG GGC-3′) binding in exon 14 and the reverse primer p865_*Glb1*_Exon16_Mus_AS (5′-GTA TCG GCC GAG GTT AAA GC-3′) binding in exon 16 of the *Glb1* gene (primers by Eurofins Genomics GmbH) and a Taq Polymerase (Invitrogen^TM^, Life Technologies Ltd.). The PCR product was sequenced at Seqlab Sequence Laboratories GmbH (Göttingen, Germany) for comparison to the WT sequence published in pubmed (NC_000075.6).

### 2.6. Enzyme Activity and Protein Determination

In cooperation with the Villa Metabolica from the University of Medicine in Mainz, the β-galactosidase enzyme activity in fibroblasts derived from *Glb1*^−/−^ and WT mice was determined using the substrate 4-methylumbelliferyl-β-d-galactopyranoside (Sigma-Aldrich Chemie GmbH, Taufkirchen, Germany) and the Hitachi fluorescence photometer (F-2000, Scientific Instruments, Schwäbisch Gmünd, Germany) as described [66]. The endogenous β-galactosidase activity was measured in nmol enzymatically hydrolyzed 4-methylumbelliferyl-β-d-galactopyranoside/mg/minute. Protein quantitation was performed in accordance with the Lowry protocol [67].

### 2.7. Experimental Design and Scoring

24 *Glb1*^−/−^ mice and 24 wildtype mice of both genders were examined for general appearance and posture, behavior and activity, and gait. Further examinations included detection of weight, parachute test [68], righting reflex [68], walking on a grid [68], hang test [69], avoidance behavior after pinching of the base of the tail, and correction of the body position after turning onto the back [68,70] (Table 1). Three male and three female *Glb1*^−/−^ mice and an equal number of wildtype mice were humanely killed at two, four, six and eight months of age (*n* = 3 mice/gender/group). The study was approved by the Local Institutional Animal Care and Research Advisory committee and permitted by the appropriate authority (LAVES, Oldenburg, Germany, permission number: 33.9-42502-04-14/1532).

### 2.8. Histology

After euthanasia, tissue samples were fixed with formalin, decalcified with EDTA (only spinal cord with adjacent vertebral bodies), and embedded in paraffin as previously described [71,72]. Four µm sections were stained with hematoxylin and eosin (HE) [48,73]. The accumulation of stored material was scored semiquantitatively in the cerebral cortex, hippocampus, thalamus, cerebellum with molecular, Purkinje, and granular cell layer and medullary center of the cerebellum, and brainstem [74]: 0 = no accumulation of ballooning vacuoles, 1 = less than 10%, 2 = less than 30%, 3 = less than 50%, 4 = less than 70%, and 5 = more than 70% of the affected neurons. Moreover, coronal sections from the thoracic, lumbar, and sacral spinal cord were investigated. Furthermore, neurons in cervical dorsal root ganglia, trigeminal ganglia, and intramural ganglia of the stomach and small and large intestine as well as tissue samples of the lung, heart, thymus, pancreas, liver, bladder, kidneys, uterus and ovaries, testes, epididymides, vesicular gland, striated muscle and femur were evaluated.

### 2.9. Immunohistochemistry

Immunohistochemistry was performed on paraffin sections applying the avidin–biotin–peroxidase complex method (Vector Laboratories Inc., Burlingame, CA, USA) with the chromogen 3.3′-diaminobenzidine-tetrahydrochloride (DAB) as previously described [75,76,77,78]. Primary antibodies directed against GFAP, CNPase, APP, MBP, Iba1, and PRX were used to investigate mice of all ages (two females and two males/time point/group) (Table 1).

Immunostainings were analyzed by counting the number of GFAP positive astrocytes, APP positive spheroids, PRX positive Schwann cells, and Iba1 positive microglia/macrophages in four high power fields (400×) using an ocular grid (1 HPF: 0.0625 mm^2^, entire area: 0.25 mm^2^, Kpl-W×/18, Carl Zeiss Meditec AG, Oberkochen, Germany) in each region of interest (ROI) in the brain. CNPase and MBP stainings were scored semiquantitatively (0 = no demyelination, 1 = mild demyelination, 2 = moderate demyelination, 3 = severe demyelination). pNF, nNF, kinesin and dynein were also examined semiquantitatively (0 = no alterations between *Glb1*^−/−^ and WT, 1 = mild differences, 2 = moderate differences, 3 = marked differences) and accumulations in swollen axons were counted.

### 2.10. Transmission Electron Microscopy

After fixation in 5% glutaraldehyde in cacodylate buffer, samples of the cerebrum, cerebellum, spinal cord, liver, and kidney were fixed in 1% osmium tetroxide, dehydrated in graded series of alcohol, and embedded in epoxy resin as described [72,73,78]. Semithin sections (0.5–1 µm) were stained with toluidine blue and examined under a light microscope (Carl Zeiss Meditec AG). From each ROI, 70 nm ultrathin sections were cut. Contrast enhancement was performed using uranyl acetate (Merck KGaA) and lead citrate. For visualization, a transmission electron microscope (Zeiss EM 10C electron microscope; Zeiss Meditec AG) was used.

### 2.11. Electrophysiology and Single Cell Electroporation

Electrophysiological experiments and single cell electroporation were carried out as described [80,81,82]. WT and *Glb1*^−/−^ mice, 3.5–five-months-of-age-old WT and *Glb1*^−/−^ mice were humanely killed and their brains were rapidly removed in an ice cold dissection solution containing (in mM): d-saccharose 120, NaCl 25, NaHCO_3_ 25, NaH_2_PO_4_ 1.25, KCl 2.5, d-glucose 25, l-ascorbic acid 0.4, myo-inositol 3, Na-pyruvate 2, MgCl_2_ 3, CaCl_2_ 0.1 (oxygenated with 95% O_2_ and 5% CO_2_ to obtain a pH of 7.4). Brains were trimmed and 200 µm thick transversal (brainstem) or sagittal (cerebellum) sections taken (Leica VT1200, Leica Microsystems GmbH, Wetzlar, Germany). Slices were incubated for 45 min at 34 °C in a recording solution containing (in mM): NaCl 125, NaHCO_3_ 25, NaH_2_PO_4_ 1.25, KCL 2.5, d-glucose 25, l-ascorbic acid 0.4, myo-inositol 3, Na-pyruvate 2, MgCl_2_ 1, CaCl_2_ 2 (oxygenated with 95% O_2_ and 5% CO_2_).

200 µm thick transversal (brainstem) or sagittal (cerebellum) sections of four-month-old WT and *Glb1*^−/−^ mice were prepared and continuously perfused with a recording solution. Recordings and electroporation were carried out at 34–36 °C under visual control of a Retiga200DC camera mounted on an upright microscope (BX51WI, Olympus SE & Co. KG, Hamburg, Germany), equipped with gradient contrast illumination. For electroporation, a glass patch pipette filled with 1 mM Alexa FluorTM 594 sodium hydrazide was pressed onto a cell and a single 10–15 ms long voltage pulse of 10–15 V was applied. Cell loading was verified with a monochromator system (Till Photonics GmbH, Gräfelfing, Germany). Slices containing loaded cells were fixed in 4% paraformaldehyde overnight. After 2× 5 min washing with PBS, slices were mounted in Vectrashield (H-100, Vector Laboratories Inc., Axxora, Lörach, Germany) and sealed under a coverslip with nail polish. Confocal image stacks of fluorescently labeled cells were obtained with a SP5 System (Leica Microsystems GmbH) using a 40× or 63× objective leading to a final voxel size of 720 or 480 nm^3^. Images were processed using ImageJ. For electrophysiological experiments, neurons of the medial nucleus of the trapezoid body (MNTB) were patched in whole-cell configuration and recorded with an EPSC10/2 (HEKA Elektronik GmbH, Lambrecht, Germany). The internal solution consisted of (in mM): K-gluconate 145, KCl 4.5, HEPES 15, Mg-ATP 2, K-ATP 2, Na2-GTP 0.3, Na2-phosphocreatine 7, K-EGTA 0.5, Alexa 488/594 0.05, adjusted with KOH to a pH of 7.2. Data were acquired with 50 kHz, and filtered at 3 kHz. Access resistance was compensated in a voltage clamp before switching to a current clamp, where bridge balance was set at 100%. For estimating the cell capacitance in the voltage clamp, data were recorded uncompensated without filtering. Data were not corrected for the liquid junction potential of about 15 mV. Data were analyzed in IgorPro and Excel. Statistical analysis was performed in GraphPad Prism (GraphPad Software Inc., San Diego, CA, USA). Significance was given at *p* < 0.05. Average data were presented as mean ± sem.

### 2.12. Lipid Analysis

Samples were weighed and homogenized using an ultra turex in 1 mL of lysis buffer (950 µL 1% Triton X-100, Tris HCl, NaCl, sodium deoxycholate, plus 50 µL protease inhibitor mix, pH 7.4). Samples were divided into three fractions for protein and lipid quantification. The first fraction was used for protein quantification with the Bradford assay [83]. The second fraction was used to isolate lipids based on a previously published chloroform–methanol-based method [84]. The third fraction was used for sphingosine and sphinganine determination and was analyzed by HPLC coupled with a fluorescence detector as previously described with minor alterations [84].

### 2.13. Lipid Raft Isolation and Western Blotting

Lipid rafts were isolated from fibroblasts as described previously [85]. All solutions, materials, and handling were performed at 0-4 °C. After two washes with phosphate buffered saline, pH 7.4 (PBS), cells were scraped into 500 µL raft buffer (1% Triton X 100 in PBS) containing protease inhibitors. Homogenization was carried out by passing the cell lysate 20 times through a 21G needle. The homogenates were then kept rocking for three hours at 4 °C. Thereafter, the lysates were adjusted to 40% *w*/*v* sucrose by adding of a volume of 80% *w*/*v* sucrose prepared in PBS. A discontinuous gradient was formed: (1 mL 80% *w*/*v* sucrose, 1 mL 40% *w*/*v* lysate, 7 mL 30% *w*/*v* sucrose, and 1 mL 5% *w*/*v* sucrose). Gradients were centrifuged at 4 °C for 18 h at 100,000× *g* using a Beckman SW40 rotor. Nine fractions were collected at 0–4 °C from bottom to top; fractions nine and eight typically contained the detergent-soluble cell pellet, and fractions one to four contained the lipid raft fractions.

Immunoblotting was performed using 50 µL of each sucrose density gradient fraction as described [84]. After denaturing in Laemmli buffer containing 100 mM DTT for 5 min at 95 °C, proteins were separated on 12% polyacrylamide gels and subsequently transferred onto PVDF membranes. The membranes were incubated in either anti-flotillin 2 antibody (Santa Cruz, 1:1000) or anti-RhoA antibody (Santa Cruz, 1:500) for one hour at 22 °C. After washing, the membranes were incubated with the secondary anti-mouse antibody conjugated to horse radish peroxidase for one hour in 2% milk (Thermo Fisher Scientific GmbH, Dreieich, Germany, 1:5000). Protein bands were visualized using SuperSignal™ West Femto maximum sensitivity western blot chemiluminescence substrate (Thermo Fisher Scientific GmbH) and a ChemiDoc XRS System (Bio-Rad Laboratories GmbH, Munich, Germany) in accordance with the manufacturer’s protocol. Quantification of protein levels was performed with ImageJ.

### 2.14. Statistical Examination and Design of Figures

For statistical analysis of non-normal distributed data (histology, immunohistochemistry, enzyme assay) Mann–Whitney U-tests were performed. Statistical significance was set at *p* < 0.05. For statistical analysis, IBM SPSS version 25^®^ for Windows (SPSS Inc., Chicago, IL, USA) was used. Graphs were designed with GraphPad Prism 7^®^ for Windows (GraphPad Software Inc.).

## 3. Results

### 3.1. Glb1^−/−^ Mice Produced Shortened Dysfunctional β-Galactosidase Due to Skipping of Exon 15

**Figure 1:**
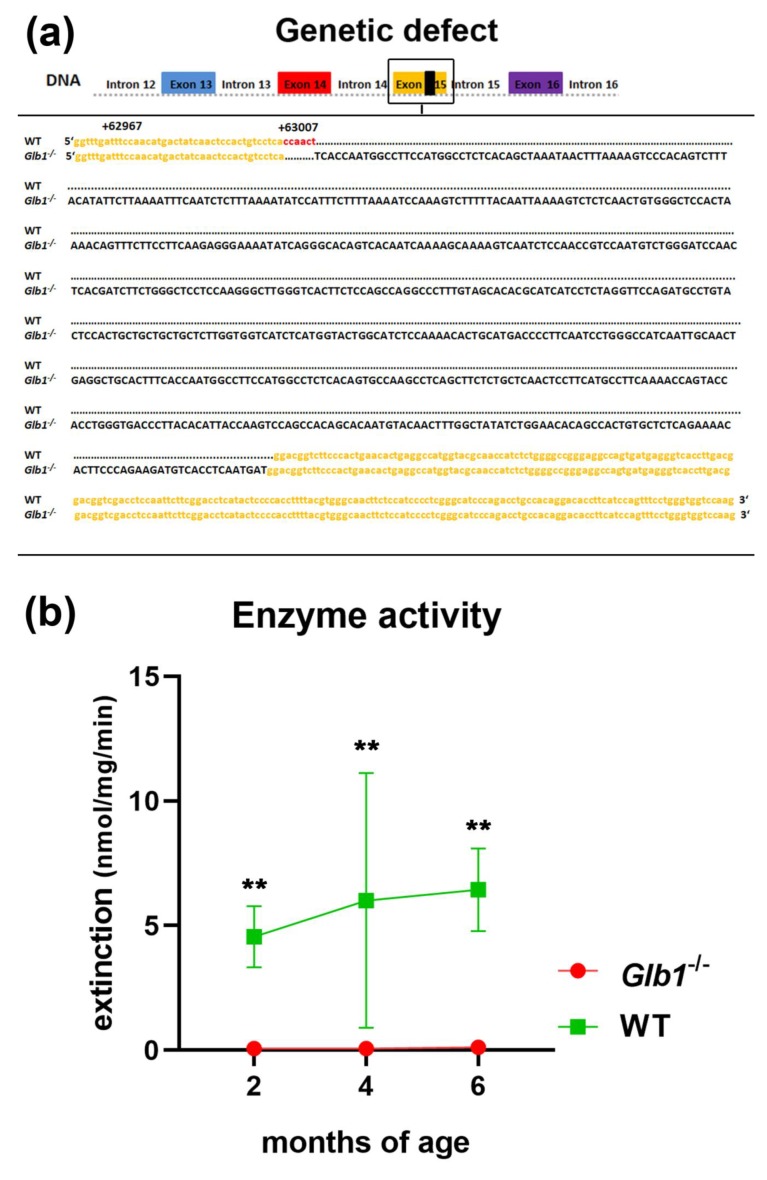
Sequence of the *Glb1*^−/−^ mice in exon 15 with the insert and enzyme activity of the β-galactosidase. (**a**) DNA sequence of parts of the exon 15 (yellow) with the knock-in (black). (**b**) Minimal β-galactosidase enzyme activity in *Glb1*^−/−^ mice compared to wildtype (WT) control mice. ** *p* < 0.01. Graphs represent mean ± SD; *n* = 2 (WT at two and four months of age, *Glb1*^−/−^ at four and six months of age), *n* = 3 (*Glb1*^−/−^ at two months of age), *n* = 4 (WT at six months of age).

Standard genotyping by PCR analysis on the *Glb1* gene demonstrated a single band of 1171 base pairs (bp) in homozygous *Glb1*^−/−^ mice (with 636 bp insert), whereas a second band of 541 bp was present in heterozygous mice. WTs revealed only one band of 541 bp. Sequencing of cDNA showed skipping of exon 15 resulting in a shortened mRNA. The 636 bp insert was introduced at the position + 1640 of the cDNA (position + 63,007 of the DNA) of exon 15 (Figure 1). At this position, 6 bp from the original DNA were deleted when introducing the insert and therefore an abbreviated protein with 562 amino acids missing 85 amino acids was assumed (647 amino acids in the WT *Glb1* protein; also see Figure S1, Supplementary Materials). The sequence of the protein was translated from the sequenced cDNA applying CLC Main Workbench (Qiagen Bioinformatics). Nevertheless, the protein sequence remains an assumption. Fibroblasts isolated from *Glb1*^−/−^ mice aged two to six months, revealed a significantly decreased β-galactosidase activity (0.03 to 0.98 nmol/mg/min) compared to fibroblasts derived from WT control mice (3.69 to 7.91 nmol/mg/min; Figure 1).

### 3.2. Glb1^−/−^ Mice Showed Increasing Neurological Disorder Starting at the Age of 3.5 to Four Months

At the age of 3.5 to four months, *Glb1*^−/−^ mice developed a neurologic disorder worsening until the age of 7.5 to eight months. This age was chosen as the terminal point due to the development of severe disease. Neurologic signs included tail flipping towards the back, mild ataxia with imbalanced steps and lateral rotation of the hind limbs and shuffling gait, difficulties in walking on a horizontal grid, a delayed correction of the body position, pulling of limbs toward the trunk in the parachute test and falling off the turned grid earlier in the hang test. Neurologic tests and scores are listed in Table 2.

### 3.3. Significant Neuronal Storage in Glb1^−/−^ Mice

**Figure 2:**
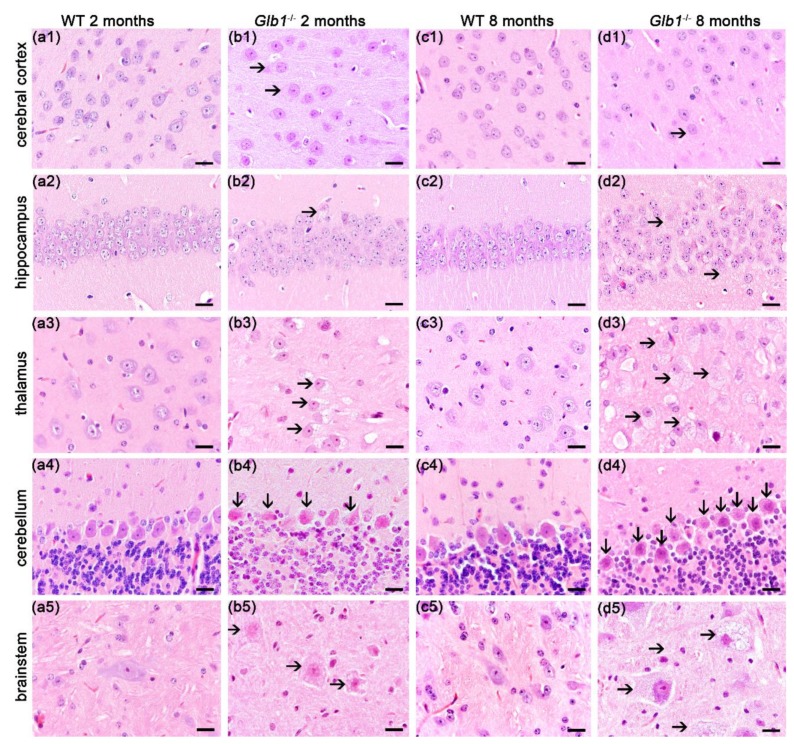
Histology of the brain in all examined areas in two- and eight-month-old mice. (**a1**–**a5**, **c1**–**c5**): Neurons of wildtype (WT) mice; (**b1**–**b5**, **d1**–**d5**): Neurons of *Glb1*^−/−^ mice with vacuolated neurons in all areas of the brain. Bars: 20 µm.

*Glb1*^−/−^ mice showed significant vacuolation and ballooning of all investigated neurons (Figure 2). The most severe lesions were present in the brainstem, thalamus as well as the medullary center and Purkinje cell layer of the cerebellum, and were also prominent in spinal cord gray matter (Figure 2). Mild vacuolation of neurons was already present in clinically unremarkable two-month-old mice (Figure 2(b1–b5)). Accumulation of the storage material in neurons continuously increased until eight months of age. At this time point, the cytoplasm of neurons in the CNS and ganglia was nearly completely filled with large vacuoles containing PAS positive material (Figure S2; Supplementary Materials).

### 3.4. Detection of Axonal Damage and Myelin Loss

**Figure 3:**
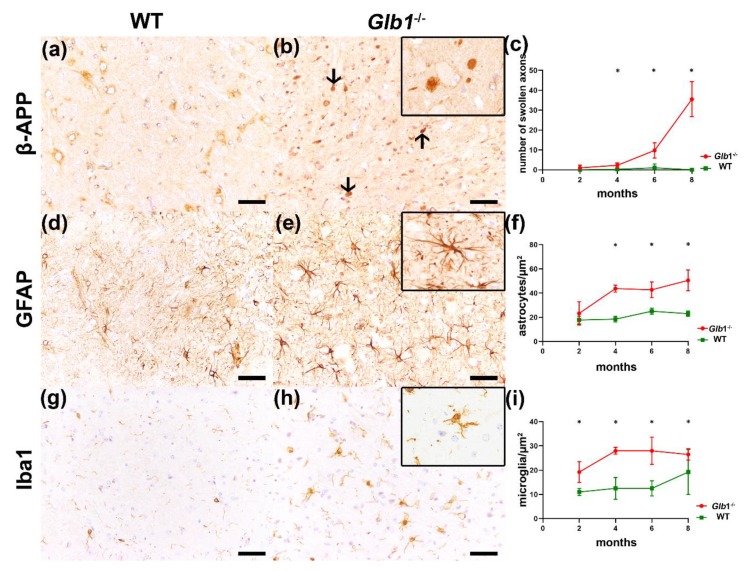
Axonal alterations, astrogliosis and microgliosis in *Glb1*^−/−^ mice. (**a**–**c**) β-APP protein in the brainstem: (**a**) Eight-month-old wildtype (WT); (**b**) eight-month-old *Glb1*^−/−^ mouse, arrows indicate β-APP positive spheroids; (**c**) quantification of β-APP positive spheroids in WT and *Glb1*^−/−^ mice from two to eight-months of age. (**d**–**f**) GFAP protein in the brainstem: (**d**) Eight-month-old WT; (**e**) eight-month-old *Glb1*^−/−^ mouse with an increase of GFAP positive astrocytes; (**f**) quantification of GFAP positive astrocytes in WT and *Glb1*^−/−^ mice from two to eight-months of age. (**g**–**i**) Iba1 protein in the brainstem: (**g**) Eight-month-old WT; (**h**) eight-month-old *Glb1*^−/−^ mouse with an increase of Iba1 positive microglia/macrophages; (**i**) quantification of Iba1 positive microglia/macrophages in WT and *Glb1*^−/−^ mice from two to eight months of age. Bars: 50 µm. red: *Glb1*^−/−^ mice, green: WT. * *p* < 0.05. Graphs represent mean ± SD; *n* = 4.

Immunohistochemistry demonstrated swollen axons, astrogliosis and microgliosis (Figure 3). β-Amyloid precursor protein (β-APP) positive swollen axons were frequently present in the medullary center of the cerebellum in all age groups, additionally in the brainstem of four to eight-month-old mice and furthermore in the cortex, the thalamus, and the cerebellar granular cell layer in eight-month-old mice (also see Figure S3, Supplementary Materials). The number of glial fibrillary acidic protein (GFAP) positive astrocytes was severely increased in the cortex, the thalamus, the granular cell layer, and the brainstem from four months of age onwards (also see Figure S4, Supplementary Materials). In the medullary center of the cerebellum, an astrogliosis was present at six and eight months of age. The brainstem showed higher numbers of ionized calcium-binding adapter molecule (Iba1) positive microglia/macrophages already at two months of age, whereas the number of Iba1 positive cells was unchanged in the thalamus, and the molecular and granular layer as well as the medullary center of the cerebellum until four months of age (Figure S5).

**Figure 4:**
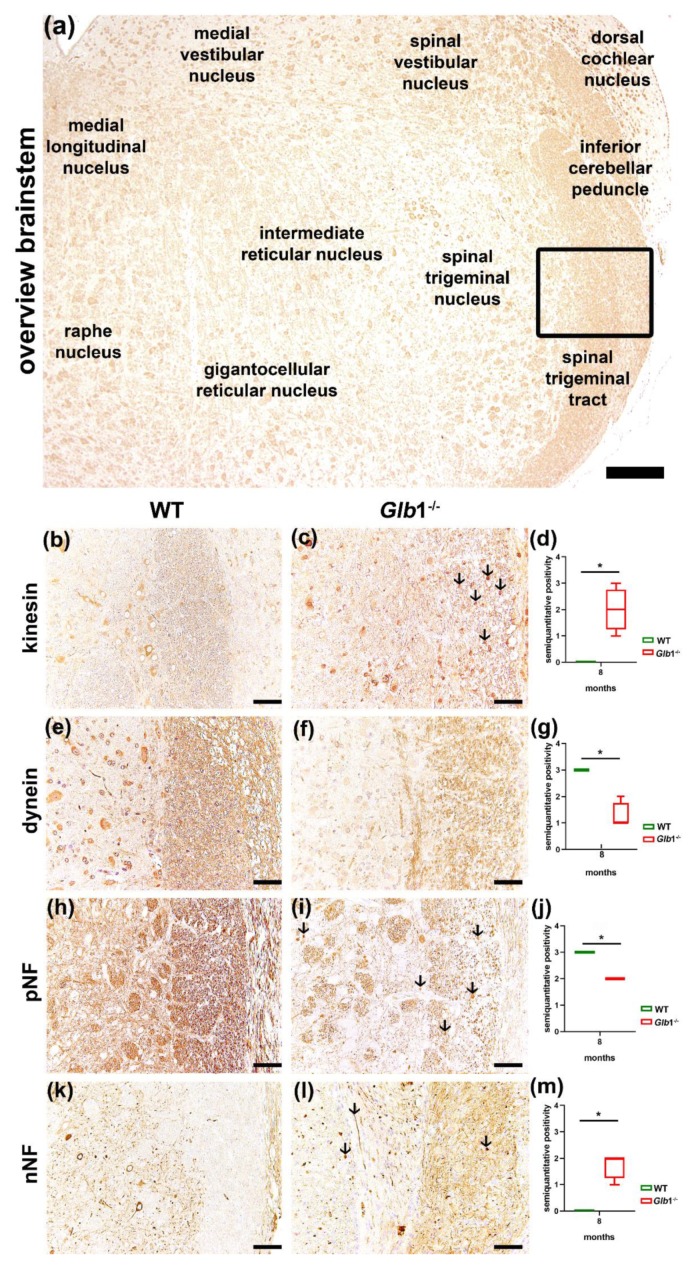
Immunohistochemistry of the brainstem with axonal damage. (**a**) Overview of the brainstem (eight-month-old wildtype (WT), kinesin). (**b**–**m**): Brainstem of an eight-month-old *Glb1*^−/−^ mouse compared to an eight-month-old WT. (**b**–**d**) Kinesin protein in brainstem axons; (**b**) eight-month-old WT; (**c**) eight-month-old *Glb1*^−/−^ mouse with several swollen axons (arrows); (**d**) quantification of kinesin positive axons in eight-month-old WT and *Glb1*^−/−^ mice; (**e**–**g**) Dynein protein in brainstem axons: (**e**) eight-month-old WT; (**f**) eight-month-old *Glb1*^−/−^ with decreased dynein-positivity; (**g**) quantification of dynein positive axons in eight-month-old WT and *Glb1*^−/−^ mice; (**h**–**j**) pNF in brainstem axons: (**h**) eight-month-old WT; (**i**) eight-month-old *Glb1*^−/−^ with decreased pNF positive axons; (**j**) quantification of pNF positive axons in eight-month-old WT and *Glb1*^−/−^ mice; (**k**–**m**) nNF in brainstem axons: (**k**) Eight-month-old WT; (**l**) eight-month-old *Glb1*^−/−^ with increased nNF positive axons; (**m**) quantification of nNF positive axons in eight-month-old WT and *Glb1*^−/−^ mice. Bars: (**a**): 200 µm; (**b**), (**c**), (**e**), (**f**), (**h**), (**i**), (**k**), (**l**): 50 µm. red: *Glb1*^−/−^ mice, green: WT. * *p* < 0.05. Box plots are used to show data; *n* = 4.

In eight-month-old *Glb1*^−/−^ mice, a significant loss of phosphorylated neurofilament (pNF) (*p* < 0.05) positive axons was found in the cerebellar and brainstem white matter. Nevertheless, the thalamus, brainstem, granular cell layer and cerebellar white matter showed a significant (*p* < 0.05) increase in pNF positive spheroids. Furthermore, a significantly (*p* < 0.05) higher number of non-phosphorylated neurofilament (nNF) positive axons were found in the brainstem of *Glb1*^−/−^ mice compared to wildtype controls (Figure 4). *Glb1*^−/−^ mice also presented an increased number of kinesin positive axons in the brainstem and in the cerebellar white matter (*p* < 0.05). Moreover, a loss of dynein staining intensity was found in the subcortical white matter, the corpus callosum, the white matter around thalamic and brainstem nuclei and the cerebellar white matter (*p* < 0.05; Figure 4).

Immunohistochemistry using antibodies directed against myelin basic protein (MBP) and 2′,3′-cyclic-nucleotide 3′-phosphodiesterase (CNPase) did not detect myelin damage (Figure S6). Furthermore, periaxin-positive Schwann cells were not present in the CNS of *Glb1*^−/−^ mice and WTs.

### 3.5. Glb1^−/−^ Mice Showed Lamellar Storage Material in Lysosomes of Soma and Axons

**Figure 5:**
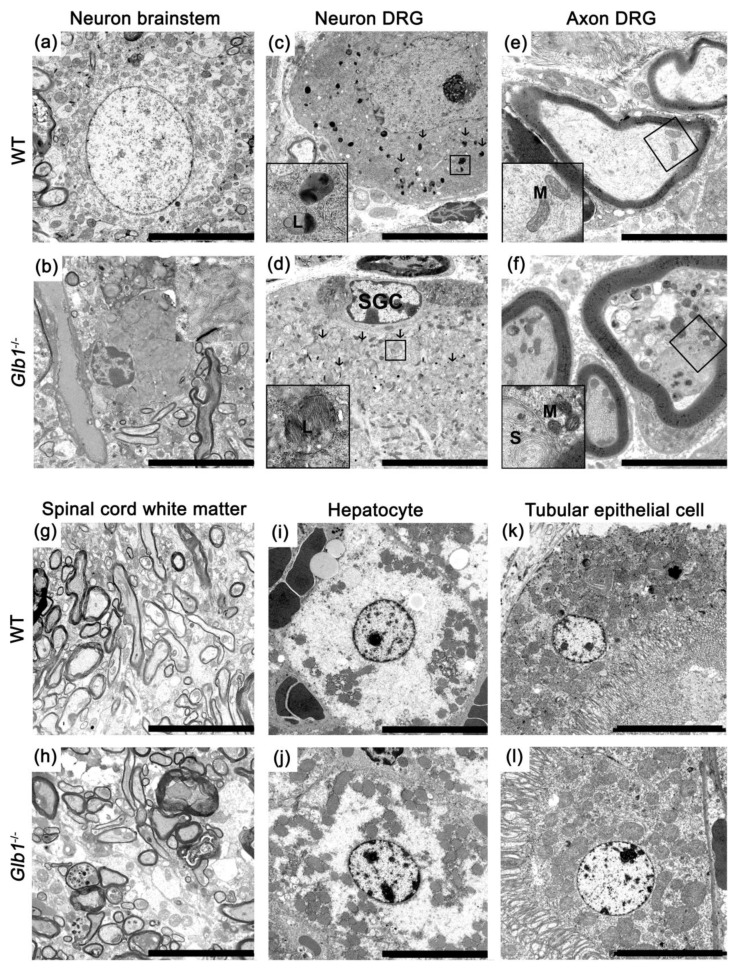
Transmission electron microscopy. (**a**) Brainstem neuron of a wildtype (WT); (**b**) brainstem neuron of an eight-month-old *Glb1*^−/−^ mouse; (**c**) dorsal root ganglion (DRG) neuron of a WT with normal lysosomes (L, insert); (**d**) DRG neuron of a *Glb1*^−/−^ mouse with lysosomal lamellated storage material (arrows and insert); adjacent satellite glial cell (SGC) without storage material; (**e**) axons from WT DRG with regularly structured neurofilaments and microtubules with few mitochondria (M, insert); (**f**) axons in a DRG from an 8-month-old *Glb1*^−/−^ mouse with lamellar storage material in lysosomes (S) and a relatively high number of mitochondria (M, insert); (**g**, **h**) myelin in the spinal cord white matter of eight-month-old WT and *Glb1*^−/−^ mice; (**i**, **j**) hepatocytes of eight-month-old WT and *Glb1*^−/−^ mice; (**k**, **l**) renal tubular epithelial cells of eight-month-old WT and *Glb1*^−/−^ mice. Bars: (**a**–**d**), (**g**–**l**): 10 µm, (**e**, **f**): 5 µm.

Neurons of the brain, dorsal root ganglia, and spinal cord of *Glb1*^−/−^ mice investigated with transmission electron microscopy (TEM) demonstrated enlarged lysosomes containing lamellar, partly concentrically arranged storage material compared to WT controls (Figure 5).

Nuclei of neurons were displaced to the margins of the cells in *Glb1*^−/−^ mice and displayed a condensed chromatin. The Nissl substance was compressed towards the nuclei. In axons, intralysosomal lamellated concentrically arranged storage material was present, displacing microtubules and neurofilaments. Additionally, dense bodies interpreted as lysosomes containing a low amount of storage material were present. Electron dense bodies were identified as lamellated, lysosomal storage material and autophagosomes within neurons (Figure S7, Supplementary Materials). Interestingly, glial cells such as astrocytes, oligodendrocytes, Schwann cells and satellite glial cells did not reveal lysosomal accumulations (Figure S8, Supplementary Materials). *Glb1*^−/−^ mice also showed a relatively high number of mitochondria in axons. Myelin sheaths were normal in *Glb1*^−/−^ and WT mice (Figure 5). No lysosomal storage was found in renal tubular epithelial cells and hepatocytes (Figure 5).

### 3.6. Neurons of Glb1^−/−^ Mice Showed Abundant Vacuolar Structures in Their Soma and Dendrites

**Figure 6:**
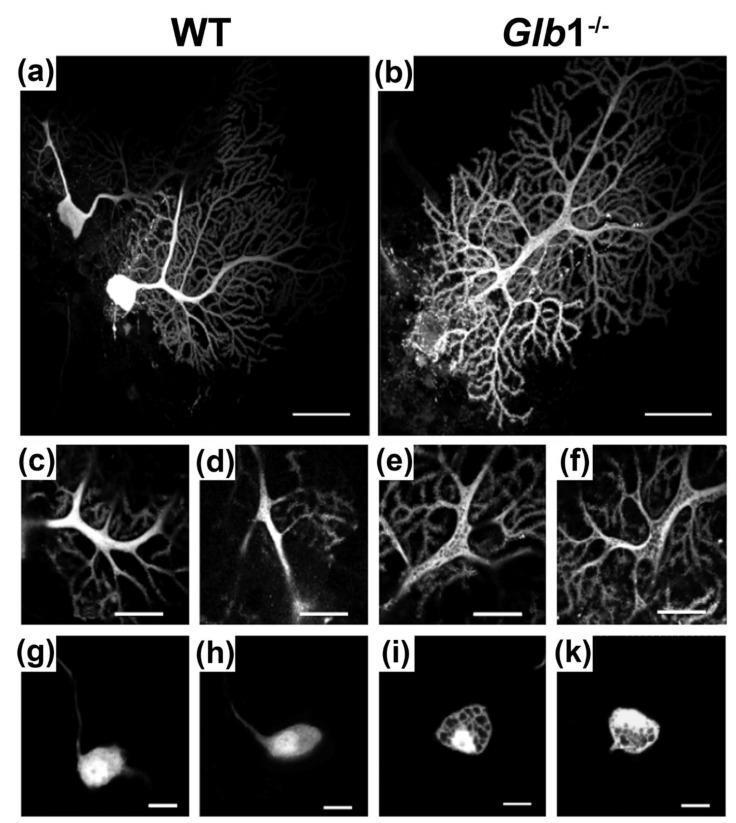
Electroporation: Single cell stainings of 3.5 to five-month-old *Glb1*^−/−^ and wildtype (WT) neurons. (**a**) Purkinje cell of a WT mouse; (**b**) Purkinje cell of a *Glb1*^−/−^ mouse; (**c**, **d**) single optical sections of Purkinje cell dendrites of the WT animal; (**e**, **f**) single optical sections of Purkinje cell dendrites of the *Glb1*^−/−^ animal; (**g**, **h**) dendrites of principal cells of the medial nucleus of the trapezoid body of the WT; (**i**, **k**) vacuolation of dendrites of principal cells of the medial nucleus of the trapezoid body of the *Glb1*^−/−^ mouse. Bars: (**a**, **b**): 60 µm, (**c**–**f**): 20 µm, (**g**–**i**, **k**): 10 µm.

The loss of *Glb1* function led to swelling of the somata by vacuolar structures. To test whether such vacuolar structures are also present in other cellular compartments, dendrites of Purkinje neurons in the cerebellum were investigated using single cell electroporation (Figure 6). The electroporated dye is supposed to evenly distribute in the cytoplasm of neurons. Since the used Alexa dye does not cross membranes to load vacuolar structures, their presence was revealed by dark, dye-less spots in the maximal projection of Purkinje neurons of *Glb1*^−/−^ mice. In single optical sections of dendrites, these vacuolar structures become clearly visible. In contrast, WT Purkinje neurons showed an even dye distribution in the maximal projection as well as in single optical sections of dendrites. This fluorescence pattern was observed in all tested cells (*Glb1*^−/−^: *n* = 5; WT: *n* = 4; obtained from two animals each). To highlight the presence of vacuolar structures also in the neurons of sensory pathways, neurons of the medial nucleus of the trapezoid body (MNTB) and auditory brainstem structure, were electroporated. Purkinje cells and neurons from the brainstem were chosen for electroporation and electrophysiological examination as histology and immunohistochemistry revealed the most prominent lesions in these regions. In *Glb1*^−/−^ mice, all neurons (*n* = 8) showed dark, dye-less spots, while control neurons (*n* = 5) of WT mice displayed an evenly distributed dye. Thus, MNTB neurons also showed the phenotypic presence of vacuolar structures in *Glb1*^−/−^ mice.

### 3.7. Biophysical Consequences of Loss of β-Galactosidase Function

**Figure 7:**
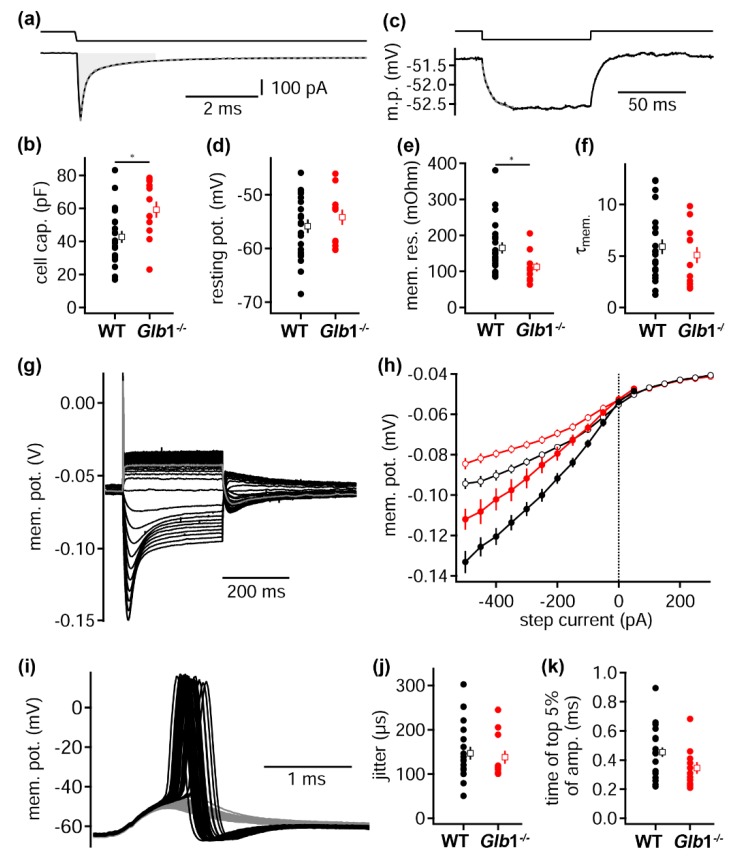
Electrophysiological characterization of G_M1_-gangliosidosis-associated lesions. Sub- and supra-threshold properties of 3.5 to five-month-old *Glb1*^−/−^ and WT neurons of the medial nucleus of the trapezoid body. (**a**) Voltage steps from −60 to −65 mV (top) induce a charging transient (bottom) used to calculate the cell capacitance (**b**). The gray shaded area indicates the region of the charge transfer (tau_weighted_ × 5) that was used to calculate the membrane capacitance. The gray dotted line represents the bi-exponential fit to the average trace. * *p* = 0.0147 (**b**) Cell capacitance of WT and *Glb1*^−/−^ MNTB neurons. Round symbols represent single cells, and open squares the average (mean ± sem). (**c**) Current injections of −10 pA (top) induce a small hyperpolarization (bottom) used to extract the membrane resistance (**e**) and the time constant (**f**). Trace represents the average of 100 repetitions; the gray dotted line is an exponential fit from start to the maximal deflection. (**d**) Resting membrane potential of WT and *Glb1*^−/−^ MNTB neurons. (**e**) Membrane resistance of WT and *Glb1*^−/−^ MNTB neurons. Symbols as in (**b**), * *p* = 0.0128. (**f**) Membrane time constant (τ_mem_) of WT and *Glb1*^−/−^ MNTB neurons. Symbols as in (**b**). (**g**) Sub- and supra-threshold voltage response to current injections. Gray trace represents first supra-threshold current injection. (**h**) Sub-threshold input–output function of WT (black) and *Glb1*^−/−^ (red) MNTB neurons extracted from voltage response shown in (**g**). Closed symbols depict the maximal voltage deflection; open symbols the voltage response of the steady state level at the end of the current injection. (**i**) Threshold current injection trigger supra- (black) and sub- (gray) threshold voltage responses. (**j**) Action potential jitter, defined as the standard deviation of the peak time of supra-threshold events of experiments as shown in (**i**). Symbols as in (**b**). (**k**) Time the sub-threshold events stay above the top 5% of the voltage amplitude. Symbols as in (**b**).

To assess the functional impact of altered membrane trafficking and intracellular vacuolar structures, electrophysiological recordings were obtained from MNTB neurons (Figure 7). This rather homogenous auditory nucleus is ideal, as the synaptic input to these globular neurons is dominated by the calyx of Held, a giant somatic synapse. Thus, somatic recordings target the compartment important for synaptic integration. From this charging transient, the cell capacitance, hence the effective cell size, was estimated. Five-times the weighted decay time of a bi-exponential fit was used to define the time course from which the transferred charge was extracted. From these data, the cell capacitance was found to be significantly larger (*p* = 0.0147) in *Glb1*^−/−^ mice (59.2 ± 16.9 pC, *n* = 12) compared to WT animals (42.8 ± 16.9, *n* = 22). The higher cell capacitance in *Glb1*^−/−^ mice implicates a greater cell surface due to cellular enlargement. Thus, our electrophysiological analysis corroborates the morphologic findings. Furthermore, the average, effective cell size of neurons is larger after functional loss of the β-galactosidase.

In a second stimulation paradigm, a −10 pA current injection led to a small hyperpolarization (Figure 7). From these data, the resting potential (baseline), the membrane time constant (exponential fit) and the input resistance were extracted. The resting potential was −54.2 ± 4.9 mV and −55.9 ± 5.8 mV in *Glb1*^−/−^ (*n* = 12) and WT (*n* = 22) neurons, and displayed no significant difference (*p* = 0.4869). The input resistance was however significantly lowered in *Glb1*^−/−^ compared to WT MNTB neurons (*p* = 0.0128). In *Glb1*^−/−^, the input resistance was 112.5 ± 42 MOhm (*n* = 12) compared to 165.9 ± 73.7 Mohm (*n* = 22) in WT neurons. The membrane time constant of MNTB neurons *Glb1*^−/−^ mice (5.09 ± 2.7 ms; *n* = 12) and WT mice (5.9 ± 3.4 ms; *n* = 22) was not significantly affected by the loss of β-galactosidase function (*p* = 0.4196). This indicates that the increase in cell membrane does not affect the integration time constants, as the input resistance compensates for the larger cell leading to a similar membrane time constant, resembling an unchanged specific membrane resistance of neurons.

To investigate further biophysical properties of these neurons, a 300 ms long square current injection with increasing amplitude was applied (Figure 7). The induced change in membrane potential was similar in *Glb1*^−/−^ and WT neurons and showed a sag response to hyperpolarizations and an onset action potential for depolarizations. In *Glb1*^−/−^ MNTB neurons, the peak and steady state hyperpolarization was significantly smaller compared to WT mice (*p* < 0.05; between −500 and −100 pA, *n* = 12 and 19 for *Glb1*^−/−^ and WT, respectively). The reduced hyperpolarization in *Glb1*^−/−^ neurons was consistent with a smaller input resistance that allowed for more injected current to dissipate. The subthreshold depolarization peak and steady state amplitudes were however not changed, indicating that no changes in the action of low voltage gated potassium channels occurred.

Since auditory neurons are exquisitely precise in the temporal aspect of input–output functions, the jitter of action potential generation at the current threshold was examined (Figure 7). The amplitude of current injections was set to generate on average 35.7% ± 13.2% and 39.1% ± 21.1% supra-threshold events in 11 *Glb1*^−/−^ and 18 WT MNTB neurons, respectively. No difference was found in the action potential waveform (height, width, de- and repolarization speed) between both genotypes. The temporal precision, jitter, was quantified as the standard deviation of the time of action potential generation. No significant difference (*p* = 0.654; *Glb1*^−/−^: 138 ± 48 µs; WT: 148 ± 62 µs) was apparent between *Glb1*^−/−^ and WT neurons. Since the time course of sub-threshold voltage deflection is indicative of the jitter [80,81], also the time the sub-threshold events spent close to the threshold was extracted. The time these events stayed within 5% of the peak amplitude was not significantly different (*p* = 0.0935; *Glb1*^−/−^: 346 ± 131 µs; WT: 455 ± 137 µs) between both genotypes.

### 3.8. Glb1^−/−^ Fibroblasts Show Lipid Accumulations and Membrane Alterations

**Figure 8:**
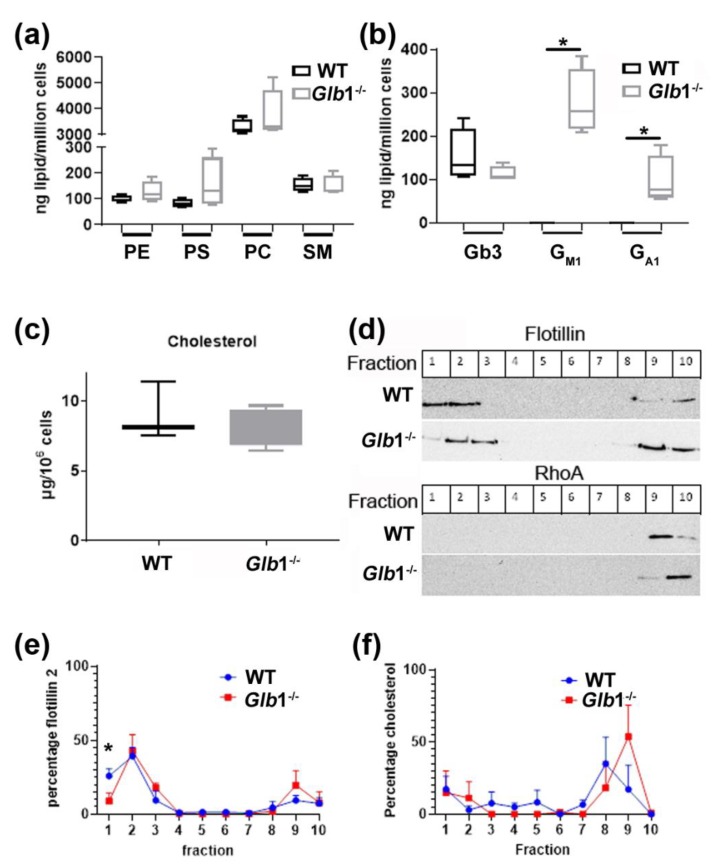
Biochemical characterization of fibroblasts. (**a**) TLC analysis of 3.5 to five-month-old *Glb1*^−/−^ and wildtype (WT) fibroblasts for phosphatidylserine (PS), phosphatidylcholine (PC) and sphingomyelin (SM); (**b**) presence of Gb3, G_M1_ and G_A1_ in *Glb1*^−/−^ mice and Gb3 in WT mice; (**c**) cholesterol concentration in WT and *Glb1*^−/−^ mice determined by HPLC; (**d**) Western blot of sucrose density gradient fractions for flotillin 2 and RhoA; (**e**, **f**) HPLC analysis of the same fractions for flotillin 2 and cholesterol distribution. * *p* < 0.05. Box plots are used to show data; *n* = 4.

Biochemical characterization of fibroblasts, cultured from *Glb1*^−/−^ and WT mice, revealed accumulations of phospholipids and significant accumulations of four glycolipids, including G_M1_, G_A1_ and two unknown glycolipids in *Glb1*^−/−^ mice. G_M1_ and G_A1_ were not detectable in WT mice. (Figure 8; also see Figures S9 and S10, Supplementary Materials). However, no change in the concentration of cholesterol between *Glb1*^−/−^ and WT cells was evident.

Interestingly, the structure of the plasma membrane was shown to be altered. Distribution of flotillin 2, RhoA and cholesterol was assessed by separating Triton X-100 cellular extracts on sucrose density gradients. The floating fractions 1–3, contained predominately cholesterol- and sphingolipid enriched lipid raft fractions, whereas the non-lipid raft fractions were retained in the lower part of the gradient (usually fractions 8–10). In WT fibroblasts, flotillin 2 was predominately found in the lipid raft associated fractions 1 and 2, with a smaller amount present in the non-raft fractions 9 and 10. *Glb1*^−/−^ cells, however, revealed a significant shift in the location of flotillin 2 towards fractions 2 and 3. Furthermore, a higher concentration of flotillin 2 was also evident in the non-lipid fractions compared to the WT. Interestingly, although no difference in the total cellular amount of cholesterol was evident, a shift in the location of cholesterol in *Glb1*^−/−^ cells could be seen from the 8th to the 9th fraction.

### 3.9. Tissue-Specific Phospho- and Glycolipid Accumulations Present in Glb1^−/−^ Mice

**Figure 9:**
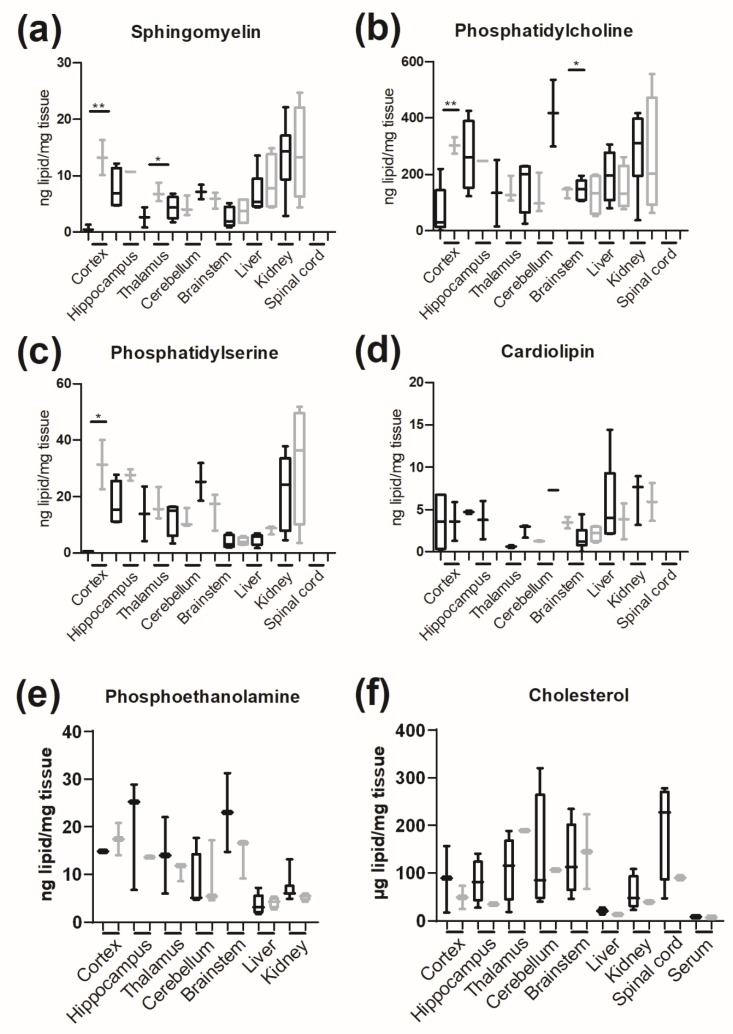
Lipid content of tissues. (**a**–**e**) TLC analysis of four-month-old *Glb1*^−/−^ and wildtype (WT) fibroblasts; (**f**) HPLC analysis for cholesterol. * *p* < 0.05; ** *p* < 0.01. Box plots are used to show data; *n* = 4.

Phospholipids and cholesterol were analyzed in five brain compartments, the spinal cord, liver, kidney and serum (Figure 9). *Glb1*^−/−^ mice demonstrated significant accumulations of sphingomyelin, phosphatidylcholine and phosphatidylserine in the cerebral cortex. Significant accumulations of sphingomyelin were also present in the thalamus, whereas significantly lower levels of phosphatidylcholine were present in the brainstem of *Glb1*^−/−^ mice. Levels of G_M1_ and G_A1_ were significantly higher in neuronal tissues in *Glb1*^−/−^ mice. Levels of G_M1_ were below the minimum detection levels in the hippocampus, brainstem, liver and spinal cord in WT mice. Similarly, G_A1_ was not found in the hippocampus, cerebellum, brainstem, liver, kidney and spinal cord of WT mice, whereas G_M1_ and G_A1_ were present in all analyzed tissues derived from *Glb1*^−/−^ mice (Figure S10, Supplementary Materials). No significant differences in sphingosine and sphinganine concentrations were detected.

## 4. Discussion

A new mouse model for human G_M1_-gangliosidosis has been characterized revealing hitherto undescribed axonal lesions and functional neuronal changes. Marked cytoplasmic neuronal vacuolization was already present in clinically unremarkable two-month-old animals, suggesting that either a low amount of storage material does not cause functional damage or potential compensatory mechanisms can prevent neurologic dysfunctions in the first months of age. Clinical signs, including movement disorders, loss of righting reflex and emaciation developed at approximately four months of age, similar to previously described mouse models [22,23,24,68,70]. Ultrastructurally, neuronal vacuoles were found to be composed of concentric and partly lamellated material as described before in mice [22,23], American black bears [21], Alaskan Huskies [48], and humans [47].

Lysosomal storage was detected not only in neuronal somata but also in axons and dendrites. The presence of lysosomes with lamellated storage material and the presence of autophagosomes might be caused by alterations in the autophagic flux, which could be an important topic in future investigations. For a better mechanistic comprehension, investigations in vitro could be performed applying fluorescence microscopy [86]. Storage in dendrites and axons was associated with abnormal distribution of APP, kinesin, dynein, pNF and nNF. nNF, pNF, dynein and kinesin are typical structural components of axons [87] and they represent typical immunohistochemical markers for axonal damage [88,89]. β-APP represents an integral membrane protein [90], which is synthesized in the soma and transported via fast kinesin-mediated axonal transport [91]. Accumulations of β-APP are commonly found in damaged axons, and antibodies against β-APP have been applied successfully for the detection of axonal lesions [88,92]. Other structural components of axons include microtubules and intermediate filaments such as actin and neurofilaments [93]. Kinesin is a motor molecule, which mediates ATP-dependent fast anterograde transport along microtubules [94,95]. Impairment of kinesin function also interferes with the fast retrograde transport mediated by dynein [87]. While membranous organelles are transported by these fast axonal transport mechanisms in both directions, cytoskeletal proteins are only moved by an independent slow anterograde transport. An altered neurofilament (NF) phosphorylation pattern is a commonly found feature in axonopathies and neurodegenerative diseases [96,97]. NFs are obligate heteropolymers composed of heavy, medium and light chains as subunits [98]. Healthy axons contain 80% highly phosphorylated NFs integrated into the axoskeleton and 20% less extensively phosphorylated NFs as a “dynamic pool” [98,99]. NF phosphorylation also controls the association of NFs with kinesin and dynein, and thereby their own axonal transport [100,101]. Thus, alterations in the amount and distribution of β-APP, pNF, nNF, kinesin, and dynein can be used as surrogate markers for axonal damage and disturbed axonal transport [75,76,88,102,103]. Furthermore, the antibodies are restricted to axons only in the CNS. Therefore, it can be assumed that their immunoreactivity indicates their axonal localization. These findings indicate that the accumulation of lysosomal storage material might influence axonal transport and neurofilament phosphorylation status [75,76,88,89,103]. A disturbance in the kinesin-mediated transport of dynein could also affect dynein distribution, because dynein transport is also partly dependent on kinesin (direct transport) [104]. A disrupted fast retrograde transport could also affect the transport of neurotrophins and removal of damaged proteins and cell organelles from the axon terminal for recycling [93]. Furthermore, *Glb1*^−/−^ mice showed variations in the distribution and amount of pNF and nNF, indicating changes in the neurofilament phosphorylation status. Altered neurofilament phosphorylation can disrupt their transport and induce local accumulations, which are commonly found in several neurodegenerative diseases [97]. Nevertheless, further examinations are needed to analyze the impact of lysosomal G_M1_ storage on axonal transport and neurofilament phosphorylation dynamics in detail. The observed astrogliosis and microgliosis in this current *Glb1*^−/−^ mouse model can be interpreted as secondary reactive lesions and have been described in human G_M1_-gangliosidosis as well [26]. However, the present findings suggest that axonopathy contributes considerably to the clinical signs found in the current model of murine G_M1_-gangliosidosis. Axonopathy in lysosomal storage diseases, particularly in G_M1_-gangliosidosis, was previously described also in a feline model [105]. In diseased cats, postnatal development of meganeurites was observed in cortical pyramidal neurons [105]. Interestingly, a myelin loss was described in different publications investigating feline G_M1_-gangliosidosis [8,50]. Contrary to cats, dogs [48] and early onset forms of human G_M1_-gangliosidosis [33,106,107], the current *Glb1*^−/−^ mice did not reveal a decreased amount of myelin.

Single cell electroporation confirmed that functional loss of β-galactosidase affects more than just the somatic compartment of neurons, since large caliber dendrites contained the same phenotype of vacuolar structures. Consequently, neither lipid metabolism in neurons with large dendrites takes place exclusively in the soma, nor do excessively generated vacuoles translocate into neuronal processes. Functionally, the major effect of *Glb1* deficiency is a reduction in the membrane resistance, since the resting membrane potential and the membrane time constant remained similar. The reduction in input resistance together with a maintenance of the membrane time constant can be explained by an increase in cell surface, where the same number of channels per surface area are incorporated. Thereby, the leak and the capacitance become larger, maintaining a balanced membrane time constant. A similar membrane time constant is potentially the main mechanism that leads to the similarity of action potentials between WT and *Glb1*^−/−^ animals. Thus, excitability is hardly affected by *Glb1* deficiency and this mechanism might also be suitable for keeping the action potential generation precise in *Glb1*^−/−^ mice. Compensating the capacitance by the input resistance represents an interesting mechanism, because decreasing the input resistance often sharpens the subthreshold depolarization and tends to make firing temporally more precise [80,81,108,109,110]. Manipulating the input resistance without causing any change in capacitance also altered the membrane time constant in these previous reports. Thus, the major membrane parameter defining the action potential generation seems to be the membrane time constant and not the input resistance. Moreover, the present *Glb1*^−/−^ mice show that at least at the outset of lysosomal storage of G_M1_, neurons successfully compensates for electrophysiological changes caused by the increased cell size. However, during the progression of the disease, the compensatory mechanisms are unable to sustain the functionality and integrity of the affected neurons and thus, the neurologic phenotype becomes evident.

Clinical signs and neuropathologic alterations in the *Glb1*^−/−^ mice are mainly caused by neuronal storage and axonal damage. Alterations in myelin amount and structure were not found when using immunohistochemistry and electron microscopy. The increased amounts of sphingomyelin, phosphatidylcholine and phosphatidylserine imply a shift from the production of gangliosides towards sphingolipids and phospholipids, due to massive ganglioside storage and consecutive inhibition of ganglioside synthesis. Sphingolipids and phospholipids are mainly produced in the endoplasmatic reticulum [111,112] and then transported to the plasma membrane via transport vesicles [111]. Increased deposition of sphingomyelin and phospholipids probably affects the structure of the cellular plasma membranes, which might influence excitability as shown by electrophysiological examination. The dry mass of myelin in situ consists of approximately 15%–30% proteins and 70%–85% lipids [113], of which cerebroside, phospholipids, cholesterol and glycosphingolipids are mostly represented [114], and complex gangliosides are also present [115]. The cellular accumulation of several phospholipids and bulky glycolipids coupled with the structural alteration of lipid rafts probably contributes to changes in neuronal function and perhaps also to the maintenance of the membrane time constant. Interestingly, a significant shift in the location of flotillin 2 towards fractions 2 and 3 revealed a redistribution of flotillin-2 in the lipid raft fractions of G_M1_-gangliosidosis cells. Flotillin 2 is a highly conserved protein isolated from caveolae/lipid raft domains responsible for signaling and trafficking of molecules within a cell [116], and abnormalities have been attributed to the pathophysiology in several lysosomal storage diseases [117]. Furthermore, it is well known that lipid rafts play a critical role in neuronal function, synapse density and morphology [118]. Alterations in the flotillin 2 distribution indicate that the membrane and in particular the organization and function of lipids rafts are altered, as has also been shown in other lysosomal storage diseases, namely Fabry disease [85]. Biochemical analyses of the current mouse model confirm that the intralysosomal storage is definitely composed of G_M1_ with a smaller amount of G_A1_. Interestingly, the increased presence of asialo-G_M1_ (G_A1_) is compatible with an alternative degradation pathway for G_M1_ and G_A1_. The presence of a higher amount of G_A1_ indicates that murine enzymes exhibit a different affinity towards degradation products, which is also described in another model for G_M1_-gangliosidosis [22] and for murine neuraminidases in a model for Morbus Tay–Sachs [44].

In the current *Glb1*^−/−^ mice, an insertion into exon 15 resulted in exon skipping and consequently generation of a shortened dysfunctional protein. Exon 15 is located in the beta domain 2 of β-galactosidase [25], which is necessary for proteolytic processing and enzyme activity, but not for the transport to lysosomes in dogs and humans [119]. In humans, various mutations of the β-galactosidase have been described [25,26,27,28,120,121,122], many of which affect mRNA splicing [123], frequently resulting in exon skipping [11] and a non-functional enzyme. Very low enzymatic activities of the murine β-galactosidase were present in the current mouse model resembling human [25,26,124], canine [9,11,65] and murine [22,23] G_M1_-gangliosidosis. Nevertheless, lack of a functional enzyme did not cause G_M1_ accumulations in the liver and kidneys. Histologically, the liver, kidney and bone lesions were also absent in the current *Glb1*^−/−^ mice, with this being similar to the adult form of human G_M1_-gangliosidosis.

Regarding the quality and onset of clinical signs, the current *Glb1*^−/−^ mice resemble previously described mouse models [22,24,68,70]. All previously published knockout mouse lines reveal very low activities of the β-galactosidase in different organs [22,23,24]. Whereas histological lesions such as neuronal vacuolation are similar in previously published mouse models and the present *Glb1*^−/−^ mice [22,23,24,74], axonal damage without myelin affection has not been described before in murine G_M1_-gangliosidosis. Lamellated storage material in neurons was also found in other *Glb1* knockout models in the CNS [22,23] and in the retina [125]. Electroretinograms of wildtype and *Glb1*^−/−^ mice did not reveal a functional impairment of retinas due to lysosomal storage [125]. Nevertheless, visual evoked potentials (VEP) were abnormal in the knockout mice, with this indicating functional and visual restrictions [125]. Electrophysiological examinations of the current *Glb1*^−/−^ mice demonstrated that the excitability of their auditory neurons is hardly affected by lysosomal storage due to the maintenance of the membrane time constant. Such studies have not been performed previously in other *Glb1* knockout models. An increase in G_M1_ and G_A1_ was found in the CNS of the current *Glb1*^−/−^ mice as well as in the CNS [24,58], optic and sciatic nerves [126] and in the retina [125] of other *Glb1* knockout mouse models. Moreover, the increase in phosphatidylserine in the CNS of the current *Glb1*^−/−^ mice resembles the findings of Heinecke et al. (2015) in the optic nerve [126]. Nevertheless, current *Glb1*^−/−^ mice also revealed accumulations of sphingomyelin and phosphatidylcholine, which might stabilize cell membrane integrity. In summary, current *Glb1*^−/−^ mice expand the existing knowledge concerning the murine disease, complementing previously described *Glb1* knockout mouse models, particularly regarding electrophysiological changes and lipid composition. The murine disease does not seem to reflect the early onset form of human G_M1_-gangliosidosis due to its late onset of clinical signs and lack of myelin loss. In contrast, the late onset of clinical signs and the fact that lesions are primarily restricted to neuronal tissue suggests that this model rather resembles the adult form of human G_M1_-gangliosidosis.

## 5. Conclusions

The detection of axonopathy and functional compensating mechanisms in this mouse model of G_M1_-gangliosidosis gives novel insights into the pathogenesis of this lysosomal storage disease, which might assist in the design of therapeutic strategies in the future. Furthermore, this model might allow investigations of axon–myelin–glia interactions in future experiments.

## Figures and Tables

**Table 1:** Antibodies used for immunohistochemistry.

1st Antibody	Clonality	Dilution	Manufacturer	2nd Antibody
Amyloid precursor protein (APP), MAB348; 22C11	Mouse monoclonal	1:2000	Millipore, Burlington, USA	Goat-anti- mouse (GAM, BioLogo, BA-9200)
2′,3′-Cyclic-nucleotide-3′-phosphodiesterase (CNPase), 11-5B		1:100		
Dynein, MMS-400P		1:25	Covance Inc., Princeton, USA	None
Non-phosphorylated neurofilament (nNF), SMI-311		1:8000	Calbiochem, Merck KGaA, Darmstadt, Germany	Goat-anti- mouse (GAM, BioLogo, BA-9200)
Phosphorylated neurofilament (pNF), SMI-312		1:8000	Sternberger Monoclonals Incorporated, MD, USA	
Glial fibrillary acidic protein (GFAP), 6F2	Rabbit polyclonal	1:1000	Dako/Agilent Technologies, Santa Clara, CA, USA	Goat-anti- rabbit (GAR, 1:200, BioLogo, BA-1000)
Iba1, PA5-27436		1:1000	Thermo Electron LED GmbH, Langenselbold, Germany	
Kinesin, K0889-100UG		1:400	Sigma-Aldrich Chemie GmbH, Taufkirchen, Germany	
Myelin basic protein (MBP), AB980		1:500	Millipore, Burlington, USA	
Periaxin (PRX), HPA001868		1:5000	Sigma-Aldrich, Taufkirchen, Germany	

β-APP is a reliable marker for axonal damage [79] and was used for a first detection of axonal alterations present in the current *Glb1*^−/−^ mice. Axonal damage of eight-month-old mice (two females and two males/group) was further characterized using antibodies directed against pNF, nNF, kinesin and dynein (Table 1). Goat-anti-mouse IgG (1:200, BA-9200) and goat-anti-rabbit IgG (GAR, 1:200, BA-1000, BioLogo Dr. Harmut Schultheiss e.K, Kronshagen, Germany) were used as secondary antibodies. However, dynein immunoreactivity was visualized by using the kit Dako REAL™ EnVision™ Detection System (K5007, Dako Denmark A/S, Glostrup, Denmark) instead of using a secondary antibody. Brains from analogous numbers of wild type age-matched mice were used as control tissue.

**Table 2:** Scoring of clinic and behavioral changes of *Glb1*^−/−^ mice.

**Tests for General Health Condition**		
**Test**	**Grade**	**Points**
General appearance	Normal posture, smooth and shiny hair	0
	Normal posture, dull and shaggy hair	1
	Mildly crooked back, dull and shaggy hair	2
	Severely crooked back, dull, shaggy and dirty hair, incontinence	3
Behavior and activity	Attentive and curious	0
	Very calm, mildly reduced spontaneous activity, unreduced induced activity	1
	Apathy, moderately reduced spontaneous activity, mildly reduced, induced activity	2
	Stupor, no spontaneous activity, little induced activity	3
Gait	Normal gait	0
	Mild ataxia, occasionally mild unsteady gait	1
	Moderate ataxia, frequently mild to moderate unsteady gait, mild staggering and stumbling	2
	Severe ataxia, frequently moderate to severe unsteady gait	3
**Neurologic Tests for the Characterization of G_M1_-Gangliosidosis**		
**Test**	**Grade**	**Points**
“Parachute” reflex	Extension and abduction of the hindlimbs, extension of the knee	0
	Mildly delayed reaction, intermitting extension of the knee	1
	Moderately delayed reaction, flexion and adduction of the hind limbs, slow movement	2
	No reaction, continuous flexing and adducting of the hind limbs	3
Grid walking	Animal does not step into mesh circuit	0
	21–30 s until stepping into mesh circuit	1
	11–20 s until stepping into mesh circuit	2
	0–10 s until stepping into mesh circuit	3
Hang test	Mouse is able to hang horizontally upside down at a grid for more than 30 s	0
	Mouse adheres for 21–30 s	1
	Mouse adheres for 11–20 s	2
	Mouse adheres for 10–0 s	3
Avoidance behavior after pinching the base of the tail	Strong reaction, squeaking	0
	Mildly delayed reaction	1
	Turning of the trunk, extension of the hindlimbs	2
	No reaction	3
Correction of the body position after turning onto the back	Immediate correction	0
	Mildly delayed correction	1
	Moderate to severely delayed correction	2
	No correction	3
**Total**		24

General health condition was determined by scoring “General appearance”, “Behavior and activity” and “Gait”. Three points in one of these categories were defined as the endpoint for the experiment, which was generally observed at the age of eight months. Parachute reflex, grid walking, hang test, avoidance behavior and correction of the body position were analyzed for a more detailed characterization of neurologic deficits during disease development of G_M1_-gangliosidosis.

## Supplementary Materials

The following are available online at https://www.mdpi.com/2077-0383/9/4/1004/s1. Figure S1: Assumed amino acid sequence of wildtype (WT) C57BL/6 (top row) in comparison to the *Glb1*^−/−^ sequence. Figure S2: PAS positive storage material in neurons. Figure S3: β-APP reactivity in WT and *Glb1*^−/−^ mice. Figure S4: GFAP reactivity in WT and *Glb1*^−/−^ mice. Figure S5: Iba1 reactivity in WT and *Glb1*^−/−^ mice. Figure S6: Intact myelin in WT and *Glb1*^−/−^ mice. Figure S7: Details of cytoplasmic structures in neurons. Figure S8: Lack of storage material in glial cells. Figure S9: Glycolipid content of fibroblasts from four-month-old WT and *Glb1*^−/−^ mice. Figure S10: Glycolipid concentration of tissues.
