# Supplementary material for: Axonopathy and Reduction of Membrane Resistance: Key Features in a New Murine Model of Human GM1-Gangliosidosis
Source: J Clin Med. 2020 Apr 2;9(4):1004. doi: 10.3390/jcm9041004 (PMC7230899; doi:10.3390/jcm9041004)
Supplement: Supplementary file 1 [file jcm-09-01004-s001.pdf]

Supplementary Materials

Axonopathy and reduction of membrane resistance: key features in a new murine model of human G_{M1}-gangliosidosis

Deborah Eikelberg, Annika Lehmbecker, Graham Brogden, Witchaya Tongtako, Kerstin Hahn, Andre Habierski, Julia B. Hennermann, Hassan Y. Naim, Felix Felmy, Wolfgang Baumgärtner, Ingo Gerhauser

Supplementary figures

```
Exon 1                                  Exon 2
5’MLRVPLCTPLPLLALLQLLGAAHGIYNVTQRTFKLDYSRDRFLKDGQPFRYISGSIHYFRIPRFYWEDRLLKMK
  ||||||||||||||||||||||||||||||||||||||||||||||||||||||||||||||||||||||||||
  MLRVPLCTPLPLLALLQLLGAAHGIYNVTQRTFKLDYSRDRFLKDGQPFRYISGSIHYFRIPRFYWEDRLLKMK
                Exon 3                                              Exon 4
  MAGLNAIQMYVPWNFHEPQPGQYEFSGDRDVEHFIQLAHELGLLVILRPGPYICAEWDMGGLPAWLLEKQ
  ||||||||||||||||||||||||||||||||||||||||||||||||||||||||||||||||||||||
  MAGLNAIQMYVPWNFHEPQPGQYEFSGDRDVEHFIQLAHELGLLVILRPGPYICAEWDMGGLPAWLLEKQ
   Exon 5                                    Exon 6
  SIVLRSSDPDYLVAVDKWLAVLLPKMKPLLYQNGGPIITVQVENEYGSYFACDYDYLRFLVHRFRYHLGNDVILF
  |||||||||||||||||||||||||||||||||||||||||||||||||||||||||||||||||||||||||||
  SIVLRSSDPDYLVAVDKWLAVLLPKMKPLLYQNGGPIITVQVENEYGSYFACDYDYLRFLVHRFRYHLGNDVILF
                                Exon 7             Exon 8
  TTDGASEKMLKCGTLQDLYATVDFGTGNNITQAFLVQRKFEPKGPLINSEFYTGWLDHWGKPHSTVKTKTLA
  ||||||||||||||||||||||||||||||||||||||||||||||||||||||||||||||||||||||||
  TTDGASEKMLKCGTLQDLYATVDFGTGNNITQAFLVQRKFEPKGPLINSEFYTGWLDHWGKPHSTVKTKTLA
                   Exon 9       Exon 10                             Exon 11
  TSLYNLLARGANVNLYMFIGGTNFAYWNGANTPYEPQPTSYDYDAPLSEAGDLTKKYFALREVIQMFKEVPEG
  |||||||||||||||||||||||||||||||||||||||||||||||||||||||||||||||||||||||||
  TSLYNLLARGANVNLYMFIGGTNFAYWNGANTPYEPQPTSYDYDAPLSEAGDLTKKYFALREVIQMFKEVPEG
                     Exon 12                             Exon 13
  PIPPSTPKFAYGKVALRKFKTVAEALGILCPNGPVKSLYPLTFTQVKQYFGYVLYRTTLPQDCSNPKIFSSPFNG
  |||||||||||||||||||||||||||||||||||||||||||||||||||||||||||||||||||||||||||
  PIPPSTPKFAYGKVALRKFKTVAEALGILCPNGPVKSLYPLTFTQVKQYFGYVLYRTTLPQDCSNPKIFSSPFNG
              Exon 14                                        Exon 15
  VRDRAYVSVDGVPQGILDRNLMTALNIRGKAGATLDILVENMGRVNYGRFINDFKGLISNMTINSTVLTNWT
  ||||||||||||||||||||||||||||||||||||||||||||||||||||||||||||||||||||||||
  VRDRAYVSVDGVPQGILDRNLMTALNIRGKAGATLDILVENMGRVNYGRFINDFK.................
                                                                   Exon 16
  VFPLNTEAMVRNHLWGREASDEGHLDGRSTSNSSDLILPTFYVGNFSIPSGIPDLPQDTFIQFPGWSKGQVW
  ||||||||||||||||||||||||||||||||||||||||||||||||||||||||||||||||||||||||
  ....................................................................GQVW

  INGFNLGRYWPTMGPQKTLFVPRNILTTSAPNNITVLELEFAPCSEGTPELCTVEFVDTPVIS*3’
  |||||||||||||||||||||||||||||||||||||||||||||||||||||||||||||||
  INGFNLGRYWPTMGPQKTLFVPRNILTTSAPNNITVLELEFAPCSEGTPELCTVEFVDTPVIS*
```

Figure S1. Assumed amino acid sequence of wildtype (WT) C57BL/6 (top row) in comparison to the *Glb1*$^{-/-}$ sequence (bottom row) encoded by the mRNA (NC_000075.6) of exon 1–16. Translation was performed applying CLC Main Workbench 7 (CLC Bio, Qiagen, Hilden, Germany). Complete interruption of the WT sequence is assumed at the end of the sequence of amino acids encoded by the mRNA of exon 14. Caused by skipping of exon 15 (dots), the sequence continues

with the amino acids encoded by exon 16 of the mRNA, which should cause an absence of 85 amino acids compared to the WT protein.

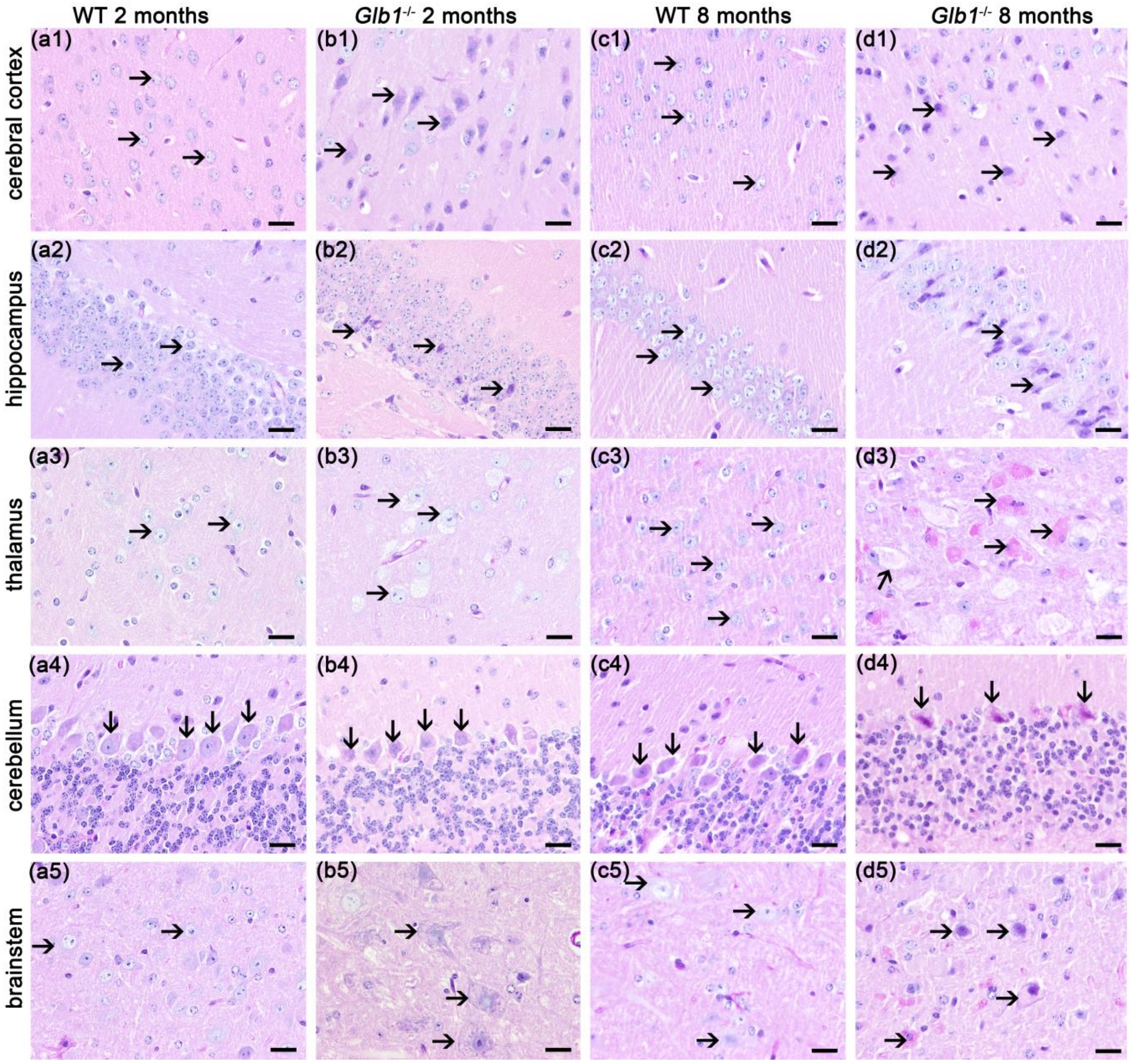

Figure S2: Neurons (arrows) in cerebral cortex (**a1–d1**), hippocampus (**a2–d2**), thalamus (**a3–d3**), cerebellum (**a4–d4**) and brainstem (**a5–d5**): Two-month-old and eight-month-old *Glb1*$^{-/-}$ mice reveal pale to bright eosinophilic material in neuronal cytoplasm (**b, d, f, h, j**). Contrary, age matched wildtype (WT) neurons (**a, c, e, g, i**) are unremarkable. Periodic acid-Schiff (PAS) reaction. Bars: 20 µm

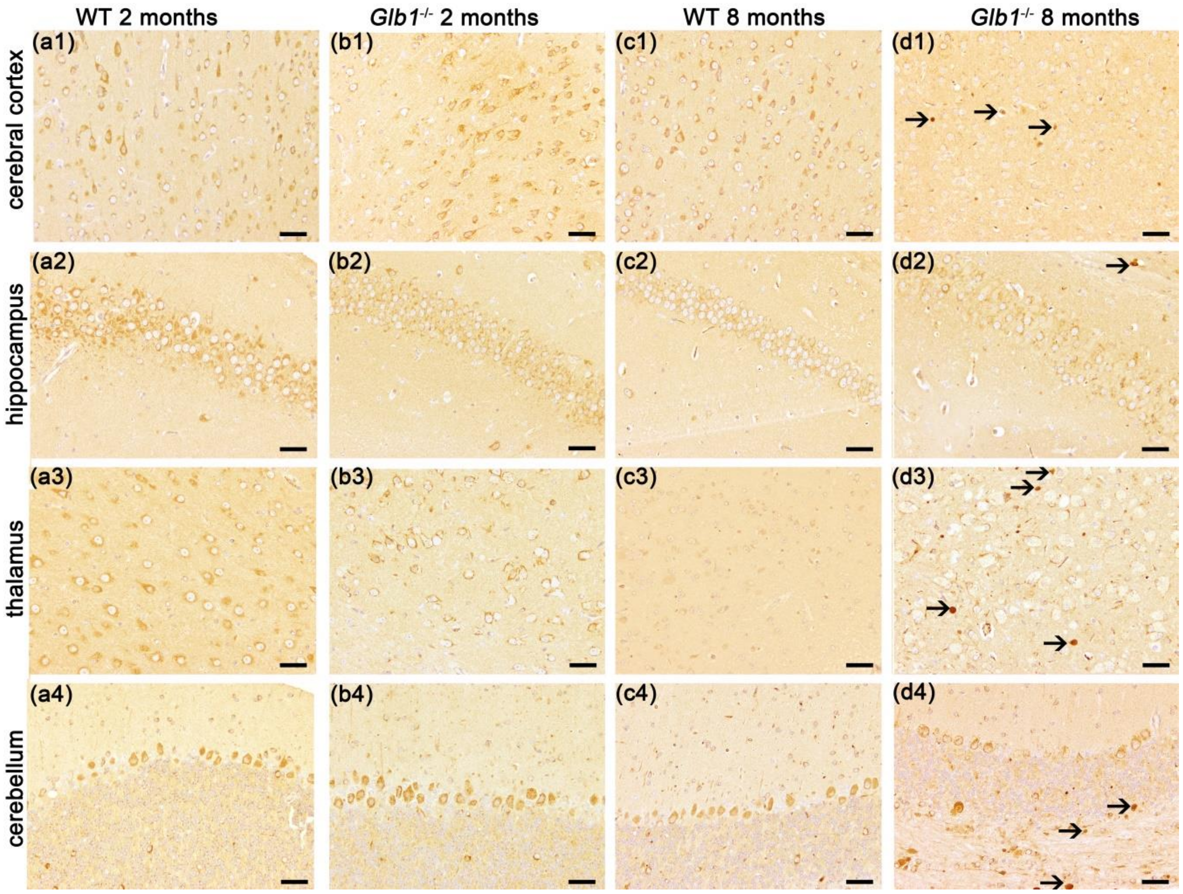

Figure S3: Significant axonal accumulations of β-APP are present in the cerebral cortex, the thalamus and cerebellum in eight-month-old *Glb1*$^{-/-}$ mice (**d1, d3, d4**) in comparison to age matched wildtype (WT) controls (**c1-c4**). Axons in two-month-old *Glb1*$^{-/-}$ mice do not show accumulations of β-APP in cerebral cortex (**d1**), hippocampus (**d2**), thalamus (**d3**), cerebellum (**d4**) and brainstem (see Figure 3, main manuscript) in comparison to an age matched WT control (**a1-a4**). β-APP positive axons (arrows) first appeared at the age of four months (data not shown). Bars: 50 μm.

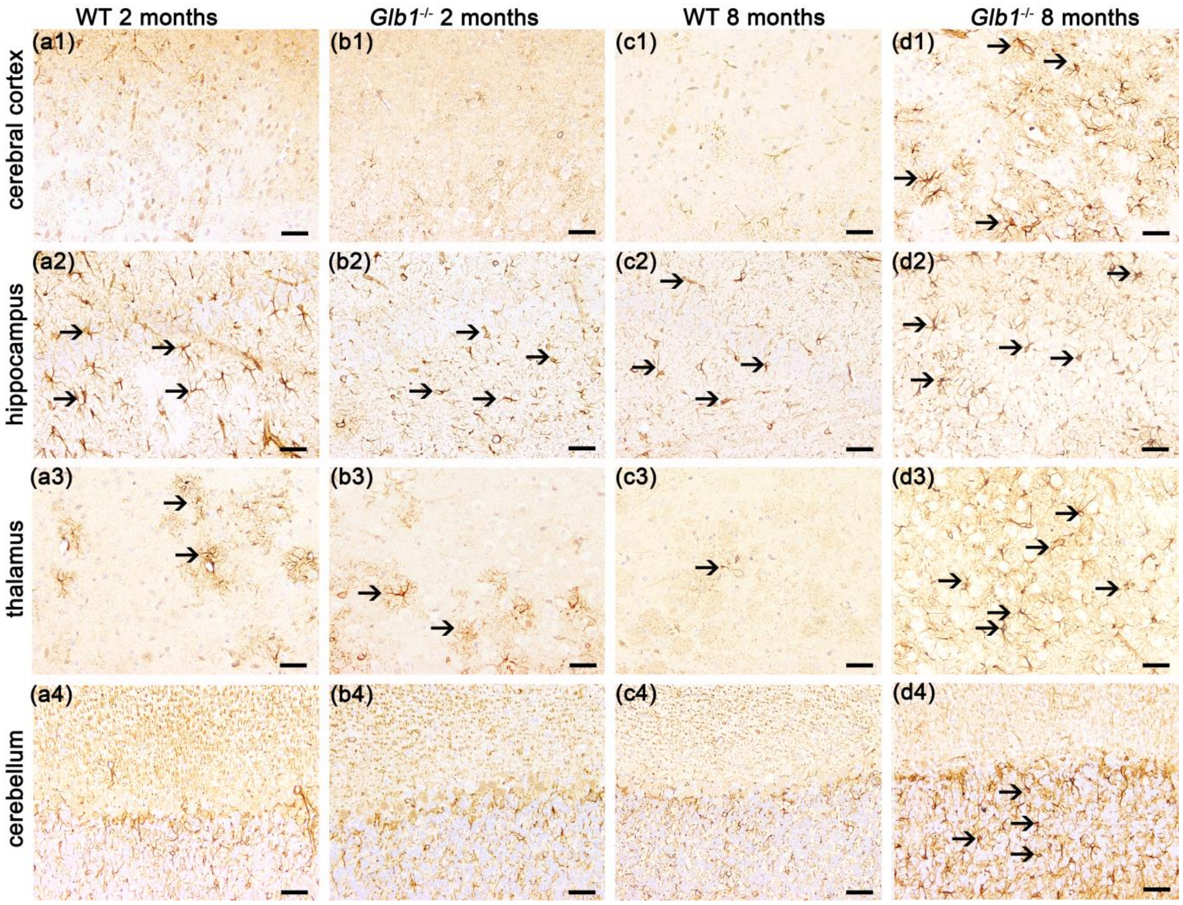

Figure S4: Astrogliosis with increased numbers of GFAP+ astrocytes (arrows) in *Glb1-/-* mice was not present in two-month-old *Glb1-/-* mice in different regions of the brain (**b1-b4**) in comparison to an age matched control wildtype (WT, **a1-a4**). From four months of age, *Glb1-/-* mice showed significantly increased numbers of GFAP+ astrocytes (data not shown). At the age of eight months, an astrogliosis was present in cortex (**d1**), thalamus (**d3**), cerebellum (**d4**) and brainstem (see Figure 3, main manuscript) in *Glb1-/-* mice (**d1, d3, d4**) compared to age matched controls (**c1-c4**). Bars: 50 μm.

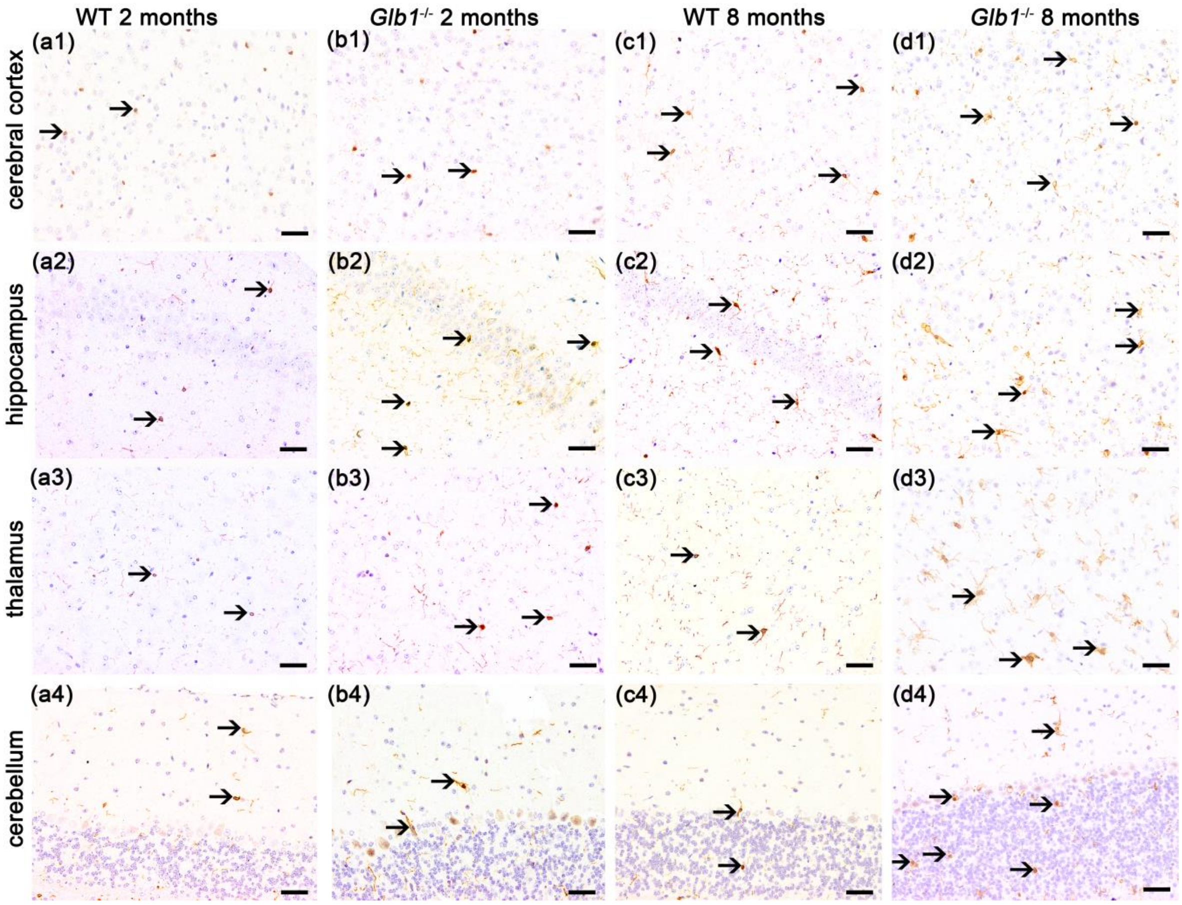

Figure S5: Increased numbers of Iba1$^+$ microglia/macrophages (arrows) were not present in two-month-old *Glb1*$^{-/-}$ mice (**b1-b4**) compared to wildtype (WT) controls (**a1-a4**). Accumulations of Iba1$^+$ microglia/macrophages developed from four months of age onwards. Eight-month-old *Glb1*$^{-/-}$ mice show significantly increased numbers of Iba1$^+$ microglia/macrophages in cerebellum (**d4**) and in brainstem (see Figure 3, main manuscript). Bars: 50 μm.

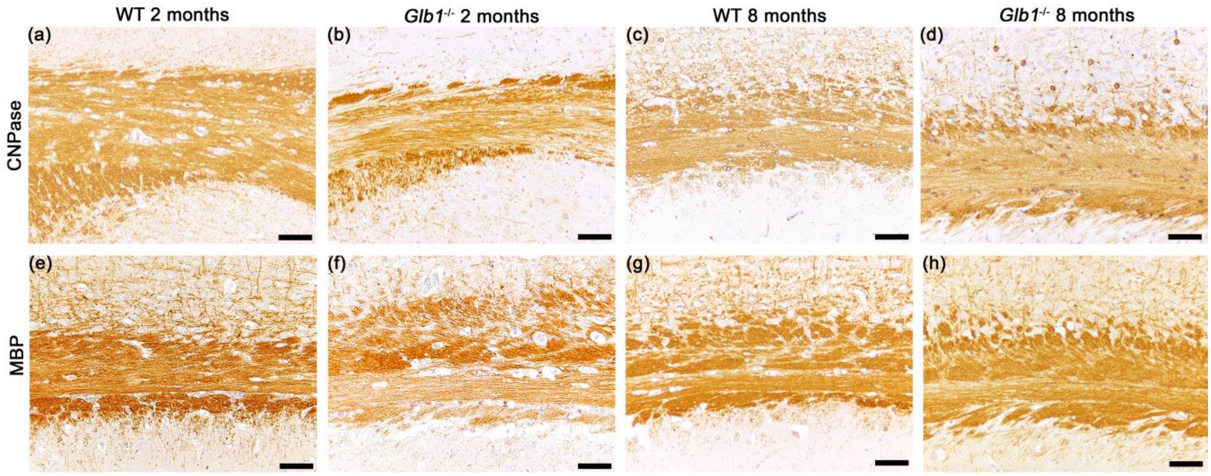

Figure S6: Corpus callosum: The amount and density of myelin is similar in a two-month-old (**a, e**), an eight-month-old wildtype (WT; **c, g**), a two-month-old *Glb1*$^{-/-}$ mouse (**b, f**) and an eight-month-old *Glb1*$^{-/-}$ mouse (**d, h**) using antibodies against CNPase (**a-d**) and MBP (**e-h**). Bars: 50 µm.

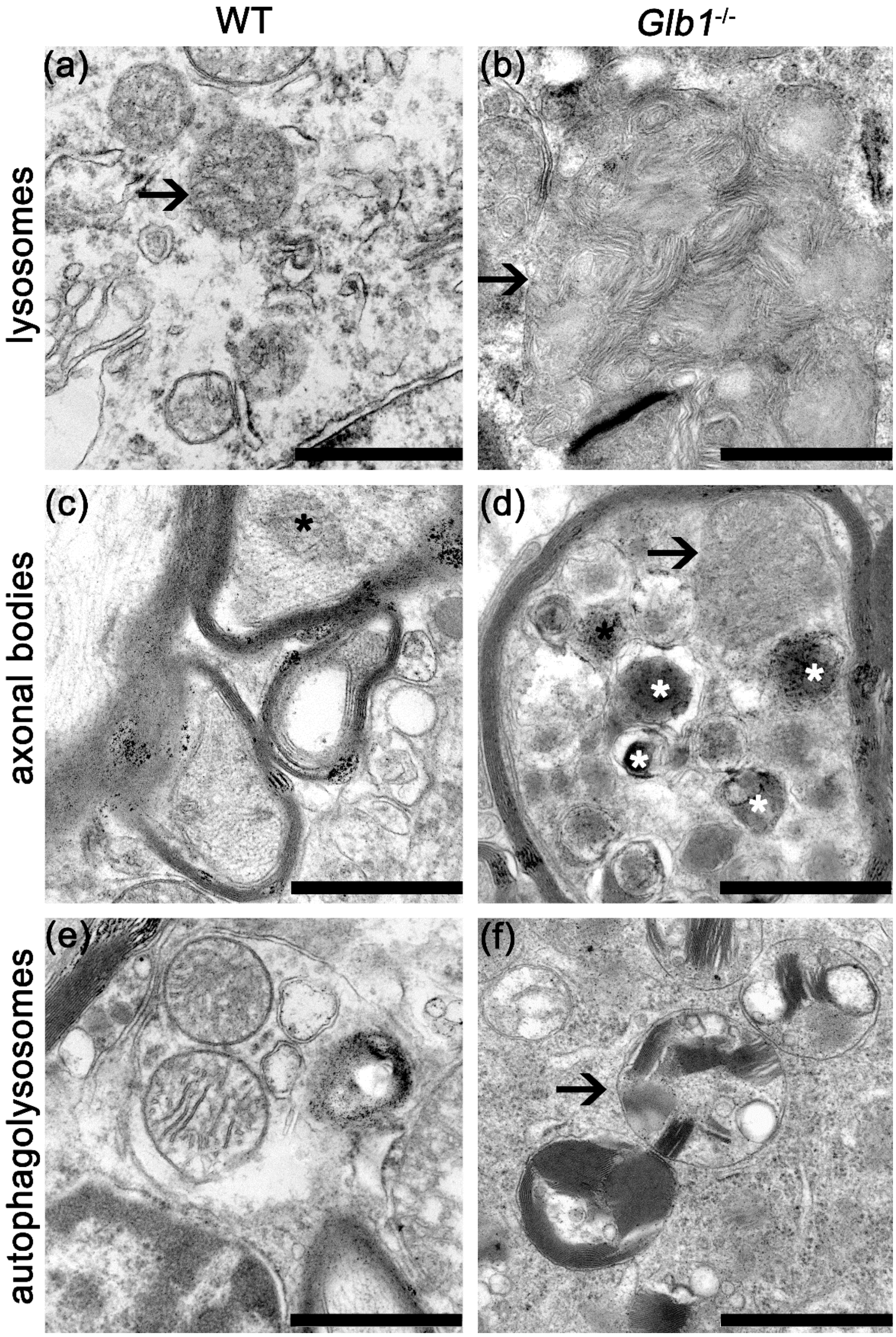

Figure S7: Details of cytoplasmic structures in neurons of the brainstem (**a, b**), axons of the spinal cord (**c, d**) and neurons in dorsal root ganglia (**e, f**) of an eight-month-old wildtype (WT; **a, c, e**) and an eight-month-old *Glb1*$^{-/-}$ mouse (**b, d, f**). Notice lamellated storage material (arrow, **b**) and an increased number of autophagosomes (stars, **d**) with an adjacent lysosome with storage material (arrow, d) in the *Glb1*$^{-/-}$ mouse. Autophagolysosomes with lamellar fragments (**f**) are also present in the

spinal cord of the *Glb1*$^{-/-}$ mouse, wheras a WT autophagolyosome (**e**) appears unremarkable. Bar: 1 μm.

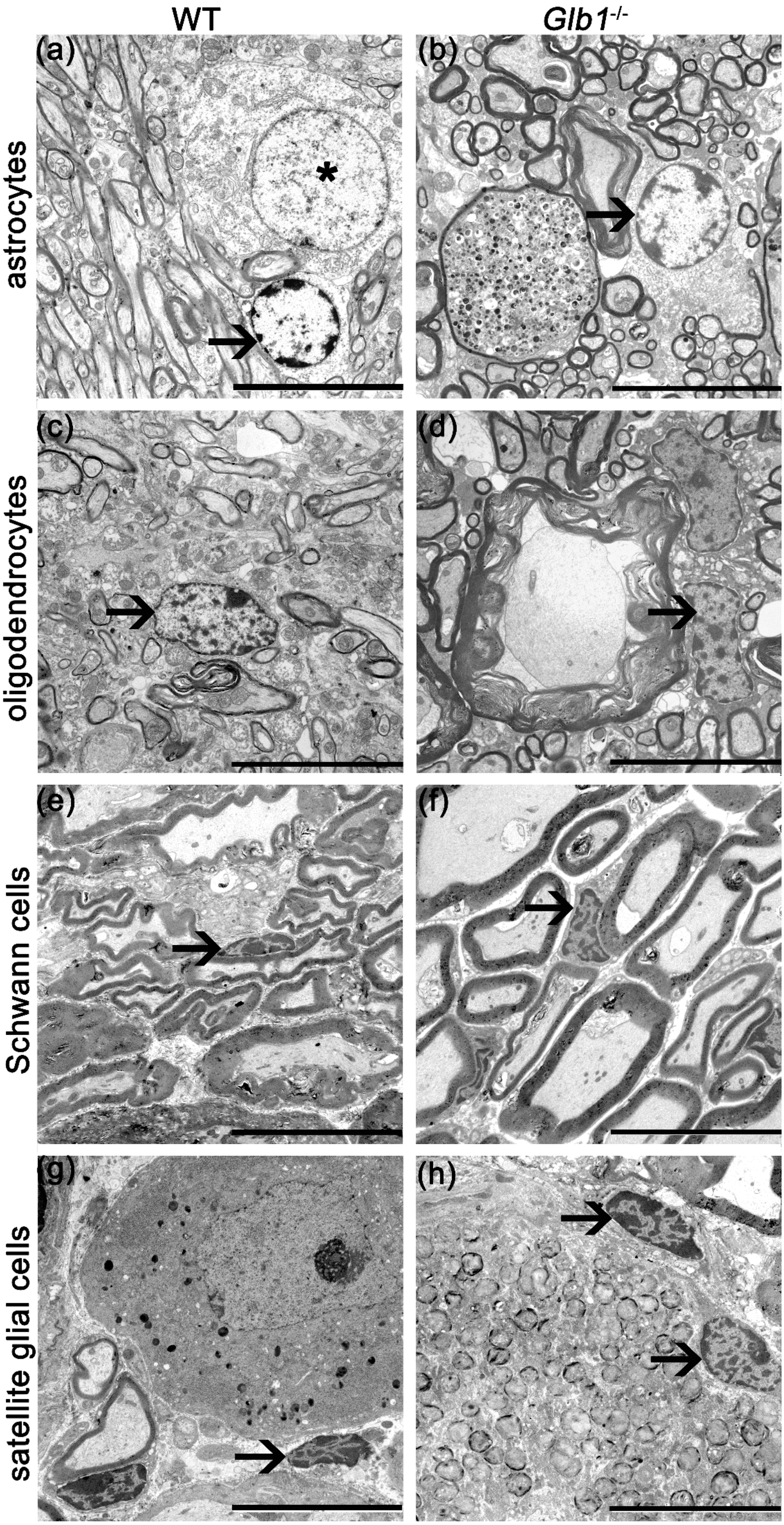
WT
Glb1$^{-/-}$
astrocytes
oligodendrocytes
Schwann cells
satellite glial cells
(a)
(b)
(c)
(d)
(e)
(f)
(g)
(h)
*

Figure S8: Intracytoplasmic storage was not present in astrocytes (**a, b**) and oligodendrocytes (**c, d**) in the brainstem of an eight-month-old *Glb1*$^{-/-}$ and wildtype (WT) mouse. Moreover, Schwann cells (e, f) and satellite glial cells (**g, h**) in dorsal root ganglia lack lysosomal storage in the *Glb1*$^{-/-}$ and WT mouse. Bar: 10 μm.

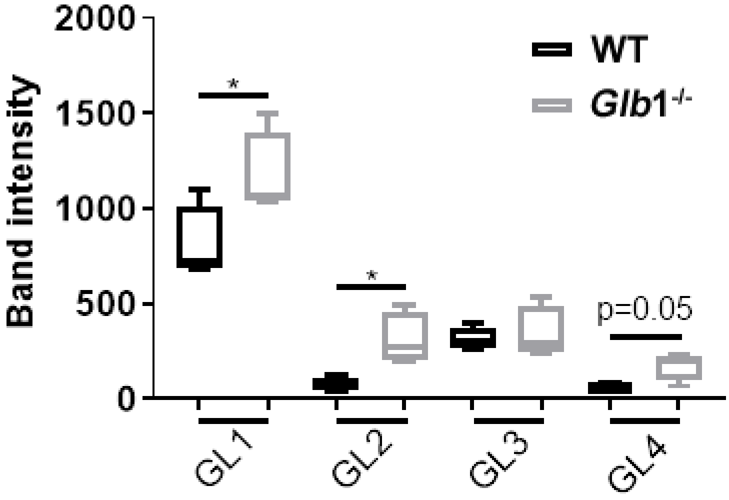

Figure S9. Glycolipid content of fibroblasts from four-month-old wildtype (WT) and *Glb1*$^{-/-}$ mice. Thin-Layer Chromatography of Lipids (TLC) analysis revealed the presence of four unknown glycolipids. Box plots are used to show data. *p <0.05; n = 4.

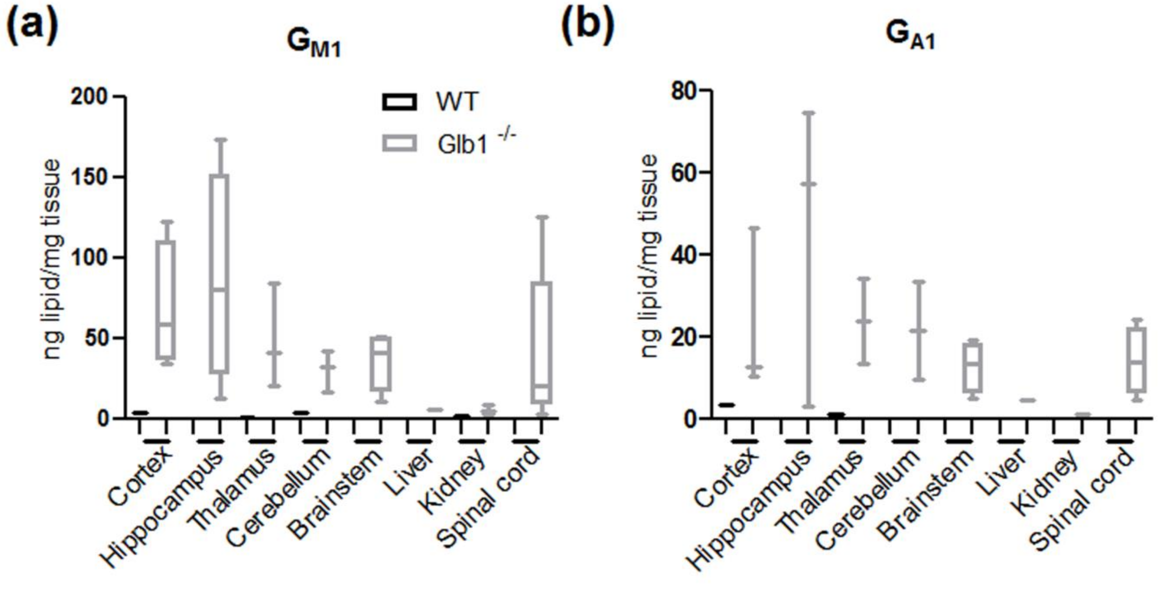

Figure S10. Glycolipid concentration of tissues. Thin-Layer Chromatography of Lipids (TLC) analysis revealed G_{M1} (**a**) and G_{A1} (**b**) in concentrations above the limit of detection (LOD) in all tissues analyzed from 3.5 to five-month-old *Glb1*$^{-/-}$ mice and in cortex, thalamus, cerebellum and kidney in 3.5 to five-month-old wildtype (WT)

mice for G_{M1} and cortex and thalamus in WT mice for G_{A1}. Box plots are used to show data. $n = 4$.
